# Wide‐Flow Aerosol Jet Printing Enables High‐Throughput, Ultra‐Low Aspect Ratio Patterning

**DOI:** 10.1002/advs.202512557

**Published:** 2025-11-03

**Authors:** Zenan Niu, Hao Yi, Yufeng Jin, Yaru Yue, Shanshan Chen, Zhixu Dong, Jia An, Chee Kai Chua, Huajun Cao

**Affiliations:** ^1^ State Key Laboratory of Mechanical Transmission for Advanced Equipment Chongqing University Chongqing 400044 China; ^2^ MOE Key Laboratory of Low‐Grade Energy Utilization Technologies and Systems School of Energy & Power Engineering Chongqing University Chongqing 400044 China; ^3^ School of Mechanical Engineering Shenyang University of Technology Shenyang 110870 China; ^4^ Centre for Healthcare Education Entrepreneurship and Research @ SUTD (CHEERS) Singapore University of Technology and Design 8 Somapah Road Singapore 487372 Singapore; ^5^ Department of Mechanical Engineering Wuhan University of Science and Technology Wuhan 430081 China

**Keywords:** functionally uniform films, high throughput, morphology control, ultra‐low aspect ratio, wide‐flow aerosol jet printing

## Abstract

Functional films have been widely utilized in flexible electronics, biosensing, and energy devices owing to their unique mechanical compliance and excellent interfacial transport efficiency. However, balancing structural precision with processing efficiency remains a key challenge in the scalable fabrication of uniform, low‐aspect‐ratio architectures across diverse material systems. Here, wide‐flow aerosol jet printing (WF‐AJP), a high‐throughput printing method capable of generating planar aerosol jets, is introduced. Reshaping the nozzle into a flattened rectangular geometry generates a collimated planar flow that enables millimeter‐scale deposition in a single pass (aspect ratio < 1:6000, thickness < 500 nm), a capability unattainable by conventional circular‐nozzle printing. Systematic cross‐scale characterization of the film formation process further reveals a delicate balance between macroscopic morphology and functional performance. A staggered deposition strategy that reduces interlayer accumulation and lowers electrical anisotropy is further implemented. Applications in conformal electrodes and skin‐interfaced sensors highlight the versatility of WF‐AJP for wearable and bio‐integrated electronics. This work establishes a structure‐controlled and scalable patterning paradigm for functional films, paving the way for future innovations in high‐throughput additive manufacturing.

## Graphical abstract

Wide‐flow aerosol jet printing (WF‐AJP) employs an anisotropic nozzle design to generate planar aerosol jets, enabling millimeter‐scale deposition of highly uniform, ultra‐low aspect ratio (<1:6000) films with substantially reduced anisotropy in functional performance. WF‐AJP establishes a structure‐controlled and inherently scalable patterning paradigm for functional films, paving the way for future advances in high‐throughput additive manufacturing.

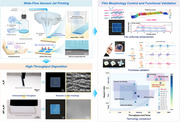

## 1 Introduction

Functional films are essential components in a broad spectrum of advanced engineering systems, spanning flexible electronics,^[^ ^1^, ^2^ ^]^ biosensing,^[^ ^3^, ^4^ ^]^ and energy devices.^[^ ^5^, ^6^, ^7^ ^]^ Low‐aspect‐ratio configurations in functional films offer mechanical compliance and excellent conformability to nonplanar surfaces, enabling seamless integration into diverse device form factors.^[^ ^8^ ^]^ Broad‐area coverage facilitates efficient interfacial transport processes, contributing to improved device performance in next‐generation lightweight and intelligent systems.^[^ ^9^ ^]^ Nevertheless, scalable manufacturing of uniform and high‐quality thin films remains challenging across diverse material systems. Conventional coating‐based techniques, including spin coating,^[^ ^10^ ^]^ blade coating,^[^ ^11^ ^]^ and dip coating,^[^ ^12^ ^]^ offer mature solutions for large‐area uniform deposition, suitable for large‐scale continuous manufacturing.^[^ ^13^ ^]^ These conventional coating methods generally lack spatial selectivity and patterning ability. Additive manufacturing (3D printing) has emerged as a versatile approach for constructing complex material architectures from micro‐ and nanoscale building blocks, offering high‐resolution patterning and broad material compatibility for functional device fabrication.^[^ ^14^, ^15^ ^]^ Despite these advantages, challenges persist in the scalable fabrication of uniform, low‐aspect‐ratio architectures,^[^ ^16^ ^]^ primarily due to the difficulty in reconciling structural precision with processing efficiency.

Aerosol jet printing (AJP) is a noncontact, digitally driven additive manufacturing technology.^[^ ^17^ ^]^ It works by atomizing functional inks into aerosols of 2–5 µm microdroplets, which are aerodynamically focused into a jet for controlled deposition of target structures.^[^ ^18^, ^19^ ^]^ In the fabrication of low‐aspect‐ratio structures, AJP offers several key advantages. First, it provides excellent vertical thickness control, enabling submicron deposition down to 100 nm, which meets the precision requirements of thin‐film structures.^[^ ^20^ ^]^ Second, its independently tunable atomization and transport modules enable precise control over material deposition. This facilitates optimization of spatial material distribution and the functional properties of the film. Aerosol droplets can reach speeds up to 50 m s^−1^, demonstrating the high‐throughput potential of the process. In addition, AJP enables in‐situ mixing of multiple functional materials within the aerosol phase.^[^ ^20^ ^]^ This supports co‐deposition of multimaterial systems and gradient compositions, significantly shortens the material development cycle for functional thin films, and provides a highly programmable technology platform for the fabrication of low‐profile and high‐performance functional films.

Nozzle‐induced geometrical constraints play a critical role in shaping the flow dynamics and determining the spatial characteristics of aerosol deposition. Since its emergence, AJP has primarily used fixed‐diameter circular nozzles to ensure consistent jet behavior and directional flexibility during in‐plane movement.^[^ ^21^ ^]^ However, this conventional design limits the trade‐off between resolution and deposition rate: high resolution requires smaller nozzle diameters, while higher deposition rates necessitate larger diameters or nozzle arrays.^[^ ^22^, ^23^ ^]^ The inverse‐cubic relationship exacerbates design limitations in objects with varying feature sizes, confining printable resolution to the nozzle diameter. While this general‐purpose strategy has enabled certain successes in microscale patterning, most studies^[^ ^24^ ^]^ report AJP achieves ≈10 µm resolution. Ma et al. achieved a minimum linewidth of 5.71 µm by ultrasound‐assisted focusing.^[^ ^25^ ^]^ Linewidth tuning can be achieved by adjusting the sheath‐to‐carrier gas flow ratio. Du et al. demonstrated continuous linewidth variation from 10 to 50 µm, accompanied by a corresponding increase in thickness from 2 to 16 µm, maintaining a nearly constant aspect ratio.^[^ ^26^ ^]^ Current AJP technology primarily enables feature size scaling^[^ ^22^ ^]^ but lacks effective control over the cross‐sectional geometry of the deposited structures. This limitation is particularly critical for low‐aspect‐ratio films: circular nozzles produce cylindrical jets that tend to form linear deposition paths, which often result in microscale voids during horizontal stacking, thereby compromising the structural uniformity and functional continuity of the film. Our preliminary experiments revealed that films printed with circular nozzles exhibit pronounced structural anisotropy (Figure S11, Supporting Information). Significant differences in electrical performance were observed along and perpendicular to the scanning direction, directly contributing to performance failure (Table S5, Supporting Information). Effective fabrication relies on the compatibility between process capabilities and the geometrical requirements of the target structure. Recognizing this, Kang et al. introduced an adaptive nozzle capable of dynamically modulating its shape during direct ink writing,^[^ ^23^ ^]^ underscoring the critical influence of nozzle geometry on structural fidelity. In aerosol‐based systems, where flow behavior is strongly influenced by nozzle‐induced constraints, tailoring the nozzle cross section offers a promising route to balance resolution and throughput in low‐aspect‐ratio film fabrication.

**Figure 1:**
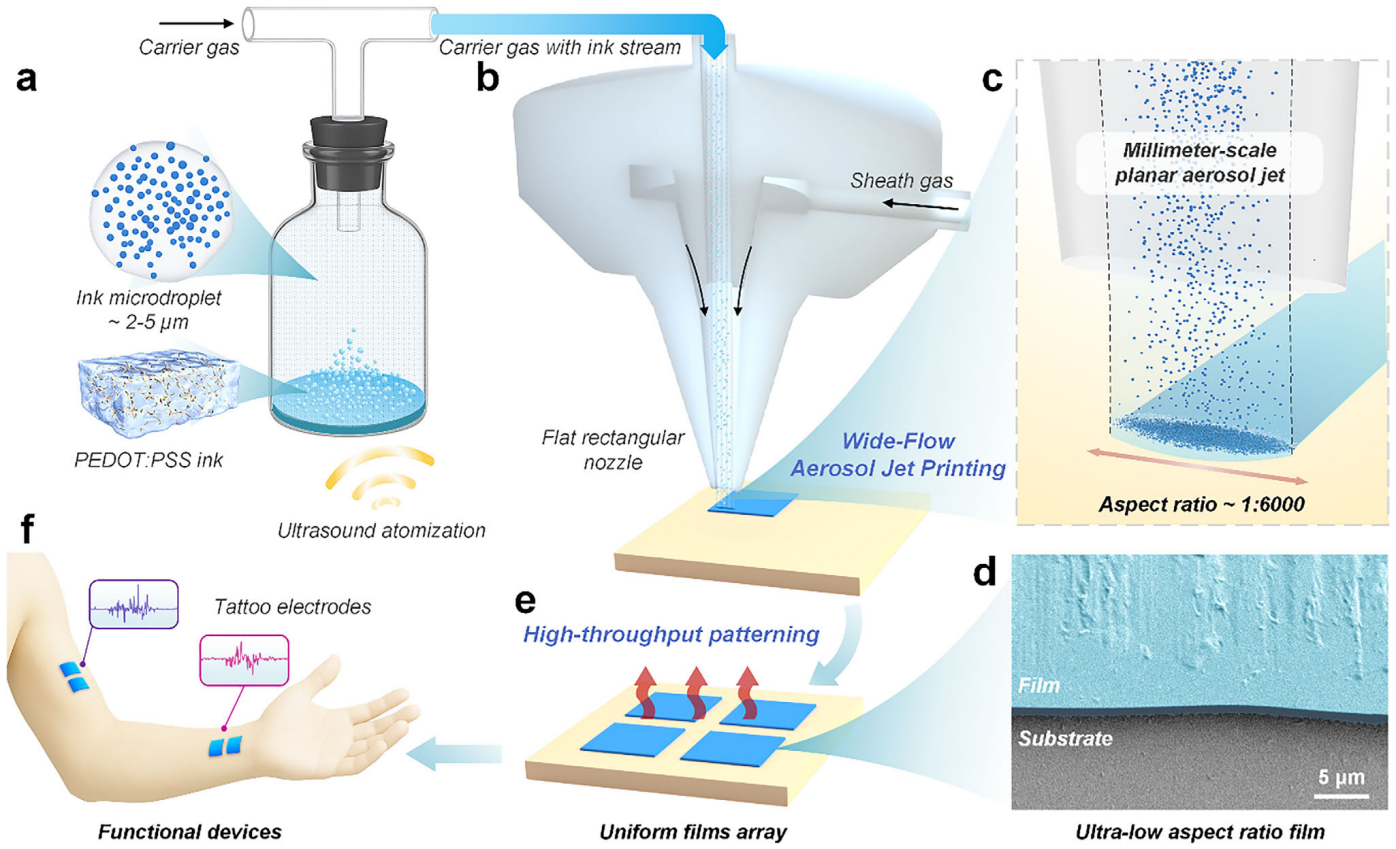
Schematic of wide‐flow aerosol jet printing for high‐throughput patterning of ultra‐low aspect ratio films. a) PEDOT:PSS ink is atomized into microdroplets (2–5 µm) using ultrasonic atomization to generate an aerosol stream. b) Aerosol stream is focused by sheath gas into a flat rectangular nozzle, forming a wide planar jet. c) Millimeter‐scale planar aerosol stream enables large‐area deposition with an aspect ratio of ≈1:6000. d) Cross‐sectional SEM image of the printed PEDOT:PSS film showing ultra‐low aspect ratio. e) High‐throughput patterning of uniform thin film arrays. f) Demonstration of functional device integration, such as tattoo‐like bioelectrodes, for wearable sensing applications.

Here, we introduce a high‐throughput patterning strategy called wide‐flow aerosol jet printing. By integrating a flat rectangular nozzle and optimizing gas flow distribution, WF‐AJP generates a stable planar aerosol stream with tunable widths up to the millimeter scale, significantly enhancing material deposition throughput (**Figure** **1**a,b). In contrast to conventional slot‐type nozzles that simply extend the outlet dimensions, the wide‐flow design incorporates internal flow field optimization to produce a collimated, planar jet with uniform velocity distribution across its width. This enables consistent, large‐area deposition with improved structural control. Using low‐aspect‐ratio films as the deposition unit, WF‐AJP effectively reduces the structural anisotropy typically observed in conventional line‐by‐line additive manufacturing and improves in‐plane structural uniformity. Experimentally, single‐pass WF‐AJP enables the formation of films with aspect ratios below 1:6000 (Figure 1c), demonstrating excellent capability for structurally controlled, wide‐field deposition. To further improve film uniformity and functional performance, we implement a staggered deposition strategy (Figure 1e). At a deposition throughput of 365.1 mm^2^ min^−1^, we obtain conductive films with a thickness of 1.9 µm, an aspect ratio of 1:5000, a surface roughness of 27.9 nm, and an electrical conductivity of 609.8 S cm^−1^, with markedly reduced in‐plane anisotropy (Figure 1d). In addition, demonstrations of conformal electrodes and skin‐interfaced sensors highlight the applicability of WF‐AJP for wearable and bio‐integrated electronics (Figure 1f), establishing a generalizable route for high‐throughput fabrication of uniform functional films.

## 2 Results and Discussion

### 2.1 Mechanism of WF‐AJP

**Figure 2:**
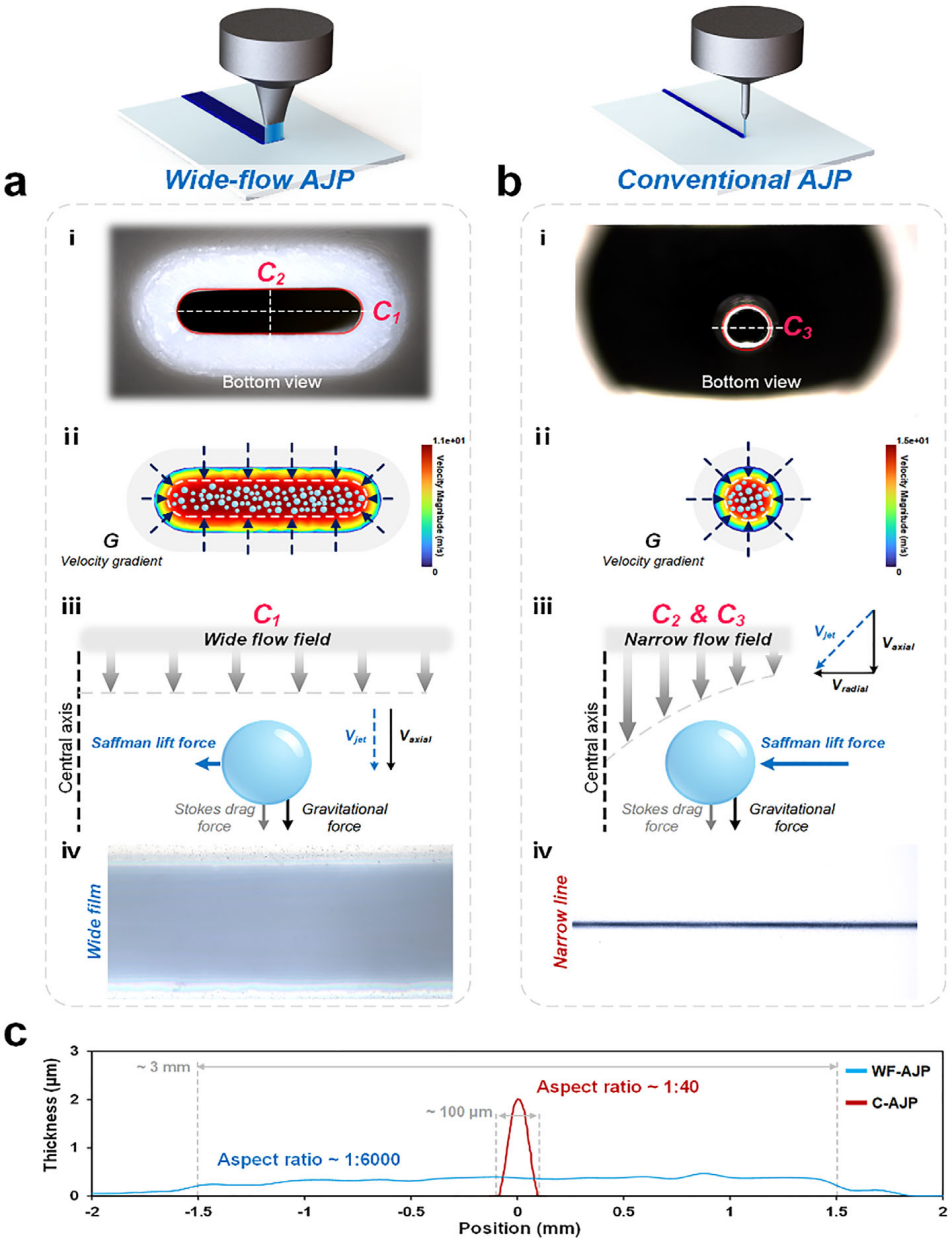
Mechanism comparison of WF‐AJP and conventional AJP. a) WF‐AJP: (i) Optical image and (ii) simulated velocity field showing a planar aerosol stream with uniform lateral flow. (iii) Droplet dynamics under wide flow, where axial velocity and Saffman lift stabilize deposition. (iv) Resulting in a wide and uniform trace. b) C‐AJP: (i) Optical image and (ii) simulation showing a narrow, axisymmetric jet. (iii) Radial velocity components induce off‐axis drift. (iv) Resulting narrow, peaked trace. c) Cross‐sectional thickness profiles of single‐pass deposited traces. WF‐AJP produces a wide film (≈3 mm width, aspect ratio ≈1:6000), whereas C‐AJP produces a narrow line (≈100 µm width, aspect ratio ≈1:40).

Wide‐flow aerosol jet printing enables functional film patterning by combining high throughput with uniform deposition. To realize this objective, it is crucial to optimize both the nozzle structure and the airflow field, minimizing turbulence and pressure losses while ensuring that the aerosol jet maintains high collimation and deposition stability over a broad area. We present a WF‐AJP printhead design that features an optimized internal flow channel geometry and a high‐throughput nozzle exit cross‐section, which together generate a stable and uniform wide‐flow aerosol jet (Figure S1, Supporting Information). As shown in **Figure** **2**, in contrast to the conventional AJP nozzle with a circular cross‐section (Figure 2b‐i and ii), the WF‐AJP printhead incorporates a rounded‐rectangular nozzle exit (Figure 2a–i and ii). The internal flow channel is designed with a continuous transition, allowing the sheath gas to uniformly encase the carrier gas, which transports the atomized droplets, thus forming a band‐like, stable aerosol jet at the nozzle exit (Figure S5, Supporting Information). This design significantly increases the lateral width of the jet, thereby enhancing material coverage efficiency and ensuring stable deposition on the substrate surface without excessive diffusion. This results in a substantial increase in printing throughput (Figure S3 and Movie S1, Supporting Information).

During the printing process, several aerodynamic forces influence the transport behavior of aerosol droplets. These forces primarily include axial Stokes drag, droplet gravity, inertial forces, and radial Saffman lift force,^[^ ^27^ ^]^ as well as pressure gradient forces. Collectively, these forces determine the flight trajectory and deposition behavior of the aerosol droplets. However, the WF‐AJP nozzle's shape introduces significant geometric anisotropy, which has a pronounced impact on the transport behavior of aerosol droplets within the flow field. To investigate how the wide nozzle affects droplet dynamics, we analyzed two orthogonal cross‐sections of the WF‐AJP nozzle for analysis: the long‐axis section (*C*_1_) and the short‐axis section (*C*_2_), with the conventional AJP circular nozzle section (*C*_3_) used as a control (Figure 2a–i, b–i). In the *C*_1_ section, the significantly increased flow channel width reduces the nozzle's confinement of the gas flow (Figure 2a–ii), leading to a gradual variation in flow velocity across the lateral direction and a sharp reduction in shear rate. Consequently, the Saffman lift force on the aerosol droplets is weakened or even eliminated (Figure 2a–iii), resulting in diminished radial focusing capability. This causes the jet to expand laterally, thus increasing the deposition linewidth (Figure 2a–iv). In contrast, the flow channel width in the *C*_2_ section remains similar to the conventional AJP nozzle, maintaining a well‐defined velocity gradient and strong shear effects (Figure 2b–ii). In this direction, Saffman lift force dominates the movement of the aerosol droplets (Figure 2b–iii), leading to significant focusing effects (Figure 2b–iv). This accounts for the marked difference in deposition linewidth between WF‐AJP and conventional AJP (Figure 2c).

The Saffman lift force acting on aerosol droplets in a shear field is expressed as Equation (1):

(1) $$F_{s}=6.46\mu {d_{drop}}^{2}\sqrt{\frac{\rho_{g}}{\mu }}•\sqrt{\left|\frac{\partial v_{g}}{\partial y}\right|}•\left(v_{g}-v_{drop}\right)$$

where *μ* is the viscosity of the gas, *d*_drop_ is the diameter of the aerosol droplet, *ρ*_g_ is the density of the carrier gas, *∂v*_g_ */∂y* is the local velocity gradient, *v*_g_ is the local gas velocity, and *v*_drop_ is the velocity of the aerosol droplet.

For the shear control in WF‐AJP at the *C*_2_ section, we can approximate the focusing behavior caused by the sheath gas to carrier gas flow rate ratio (*χ = Q*_s_ */Q*_c_) using the focusing model based on the Poiseuille flow assumption from conventional AJP. The effective focusing radius can be calculated using Equation (2):

(2) $$R=\tau \sqrt{1-\sqrt{\frac{x}{x+1}}}$$

where *τ* represents the radius of the flow channel in the short‐axis direction. Although this model is derived from the conditions of the *C*_3_ section in conventional AJP, it is still applicable to the local approximation structure of the short‐axis direction in WF‐AJP. This model effectively reflects the control trend of sheath gas compression on the degree of jet contraction.

The overall transport behavior of aerosol droplets can also be described by the dimensionless Stokes number (*Stk*), defined in Equation (3):

(3) $$Stk=\frac{\tau_{p}U}{L}$$

where *τ*_p_ is the relaxation time of the droplet, *U* is the airflow velocity, and *L* is the characteristic length. Like conventional AJP, WF‐AJP maintains comparable *Stk* values in the *C*_2_ section, allowing aerosol droplets to follow the airflow effectively. However, in the *C*_1_ section, the flow channel size is expanded, increasing *L*, which leads to a relative increase in the *Stk* value. This results in a decreased ability for aerosol droplets to follow the flow, promoting lateral expansion and distribution scattering, thereby achieving wide‐area deposition.

**Figure 3:**
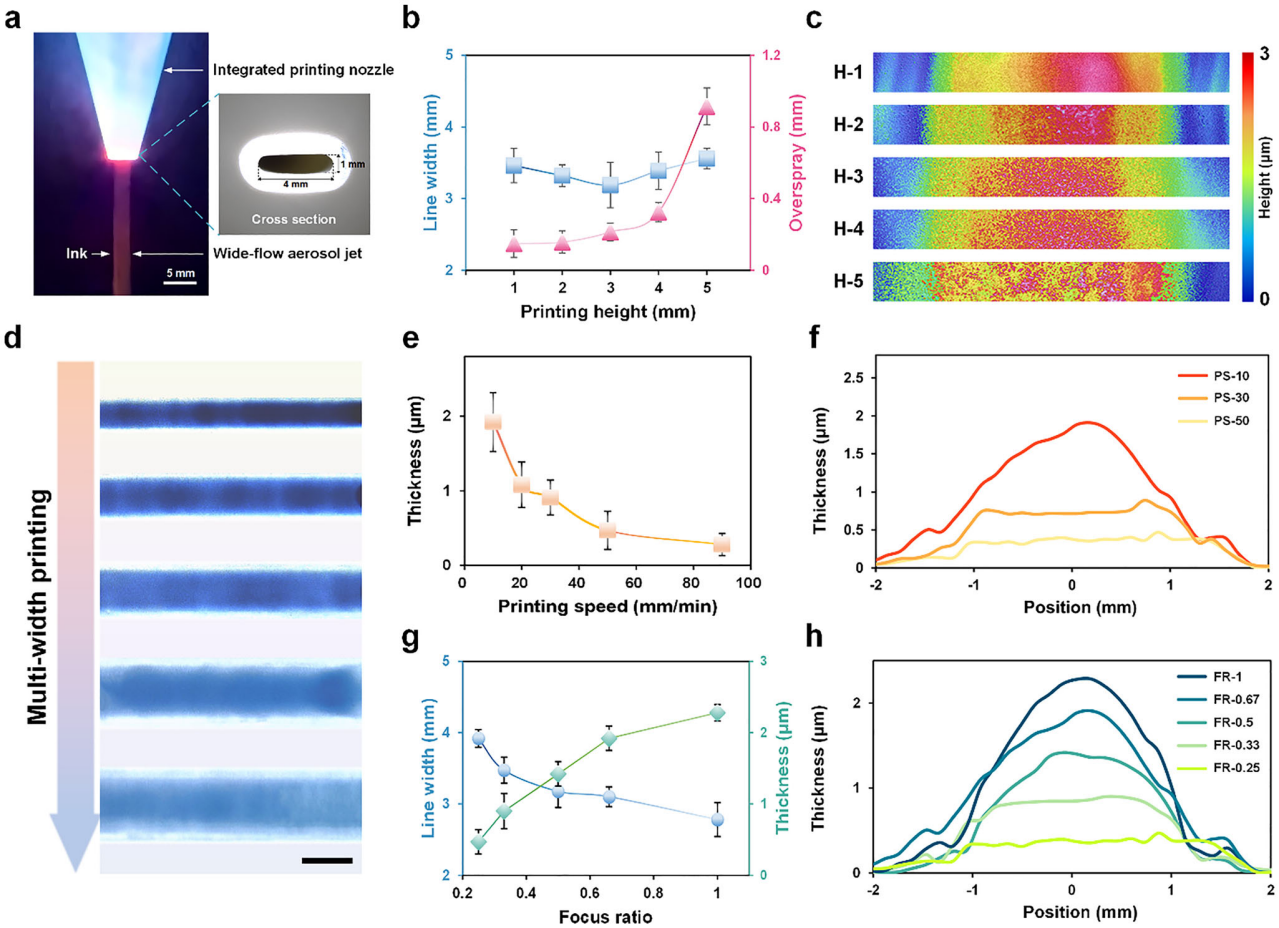
WF‐AJP deposition and process control. a) Fast camera image of the wide‐flow aerosol jet and the corresponding nozzle outlet cross‐sectional view. b) The effective linewidth and overspray width at different printing heights (SGFR = 600 sccm, CGFR = 600 sccm, *V* = 20 mm min^−1^). c) White light interference images of different printing height lines. d) Light microscope images of multiwidth printed lines. e,f) White light interference measurement results of deposition thickness (e) and cross‐section shape (f) at different printing speeds (SGFR = 600 sccm, CGFR = 600 sccm, *H* = 1 mm). g,h) White light interference measurement results of effective linewidth, deposition thickness (g) and cross‐section shape (h) under different printing focus ratios (*H* = 1 mm, *V* = 20 mm min^−1^).

In conclusion, WF‐AJP achieves spatial control over aerosol droplet dynamics through anisotropic design of the nozzle geometry. While maintaining excellent focusing performance in the short‐axis direction, the long‐axis direction releases the constraints on the jet, significantly expanding the deposition width (**Figure** **3**a). This focusing control mechanism, based on the differences in force field structures, is one of the core advantages of WF‐AJP in achieving high‐throughput and high‐uniformity functional film patterning.

### 2.2 High‐Throughput Deposition and Process Control

The primary objective of WF‐AJP is to enhance both printing throughput and deposition uniformity, which are crucial for its application in large‐area, high‐performance film manufacturing. Achieving this goal necessitates a clear understanding of the balance between deposition expansion and uniformity. Uncontrolled increases in throughput could lead to inconsistencies in film morphology, thickness, and functional properties, ultimately resulting in structural failure. The controllability of WF‐AJP structures is governed by key printing parameters, such as total printing throughput, nozzle‐to‐substrate distance, scanning speed, sheath‐to‐carrier gas flow rate ratio, and substrate temperature. These factors affect the aerodynamic characteristics of the aerosol flow, droplet‐substrate interactions, and solvent evaporation dynamics, which ultimately determine the morphology and functional stability of the printed structure.

Figure 3b illustrates the effect of the printing height (*H*) on the effective deposition linewidth (*LW*) and overspray width (*OW*). Experimental results indicate that increasing the nozzle‐to‐substrate distance from 1 to 5 mm, the effective deposition linewidth changed slightly (3.19 ± 0.07 to 3.56 ± 0.14 mm), with an overall fluctuation range of ≈11.6%. In contrast, the measured overspray width showed a significant increase, from 0.15 ± 0.07 to 0.91 ± 0.10 mm. At lower printing heights (1–2 mm), the diffusion of aerosol droplets was limited by the shorter flight distance, resulting in better focusing of the aerosol jet and more accurate droplet deposition. As the printing height increases (3–5 mm), aerosol droplets traveled longer paths, leading to increased turbulence and Brownian diffusion effects,^[^ ^28^ ^]^ which caused a large expansion of the overspray region (>600%). Notably, despite the significant change in the overspray area, its impact on the main jet flow width and the effective linewidth of the core region remains relatively limited (Figure 3c). These results suggest that, in the WF‐AJP process, the overspray phenomenon primarily arises from peripheral diffusing droplets rather than a substantial change in the size of the main jet.

The relative motion speed (*V*) between the nozzle and the substrate determines the deposition rate of aerosol droplets per unit area in WF‐AJP. As shown in Figure 3e, increasing the nozzle movement speed (from 10 to 90 mm min^−1^) significantly reduces the deposition layer thickness (Table S2, Supporting Information), decreasing from 1.92 ± 0.41 to 0.28 ± 0.12 µm, indicating a strong negative correlation between them. The comparison shows that nozzle speed primarily affects longitudinal stacking rather than lateral diffusion (Figure 3f; Figure S6, Supporting Information). These results suggest that excessively high scanning speeds can increase deposition throughput but may reduce the aerosol droplet deposition density, affecting the structural functionality. Conversely, excessively low speeds may result in solvent accumulation, destabilizing the structure.^[^ ^29^ ^]^

The wide‐flow aerosol jet channel design significantly enhances dimensional flexibility, allowing for accurate deposition across a broad range of feature sizes. With the carrier‐gas flow rate held constant, decreasing the focusing ratio from 1.0 to 0.25 results in an increase of the deposition linewidth from 2.78 ± 0.24 to 3.92 ± 0.12 mm, while the deposition thickness decreases substantially from 2.28 ± 0.11 to 0.47 ± 0.17 µm (Figure 3g; Figure S7 and Table S3, Supporting Information). Figure 3h compares the deposition cross‐sections at different focus ratios (0.25, 0.33, 0.5, 0.67, and 1). This trend illustrates the dynamic control of the aerosol jet size through airflow focusing. As the focus ratio increases, the sheath gas flow speed increases significantly, resulting in higher gas shear rates. This enhances the Saffman force acting on the aerosol droplets,^[^ ^27^ ^]^ causing greater radial contraction of the aerosol jet, altering the flux distribution in the horizontal direction, and leading to differences in deposition linewidth and thickness for the same printing throughput. Notably, for the WF‐AJP nozzle, the velocity gradient along the long axis of the flow field is lower, while the gradient in the short axis (narrower region) is higher. This results in finer deposition features along the short axis, while the long axis is more prone to expansion. WF‐AJP technology achieves a balance between improving manufacturing throughput and maintaining good accuracy in the short‐axis direction.

In terms of manufacturing capabilities, WF‐AJP technology can achieve variable linewidth deposition by simply adjusting key process parameters (Figure 3d). Compared to conventional printing processes, WF‐AJP offers greater flexibility in controlling both the size and the distribution characteristics of the deposition area, ensuring uniformity while maintaining high throughput. In particular, the anisotropic nozzle design enables a transition of the deposition profile from a Gaussian‐like to a flat‐top distribution, which underpins its ability to produce stable linewidth variations across a wide range. This tunability, supported by quantitative correlations between process parameters and structural metrics, defines a clear process window that ensures reproducibility and establishes tolerance ranges for reliable WF‐AJP fabrication.

### 2.3 Thermal Effects on Film Morphology and Defect Formation

Temperature significantly affects the microstructure of PEDOT:PSS films by altering the solvent evaporation rate, which governs the reorganization and crystallization of PEDOT chains, thereby influencing phase separation and electrical properties. Previous studies have shown that moderate heating (below 50 °C) promotes the uniform distribution of PEDOT cores, optimizes the degree of phase separation, and enhances the continuity of conductive pathways.^[^ ^30^ ^]^ However, excessive temperatures may exacerbate PSS phase separation, leading to the aggregation of PEDOT particles, reducing conductivity, and inducing internal stress accumulation within the film (Figure S8, Supporting Information).

**Figure 4:**
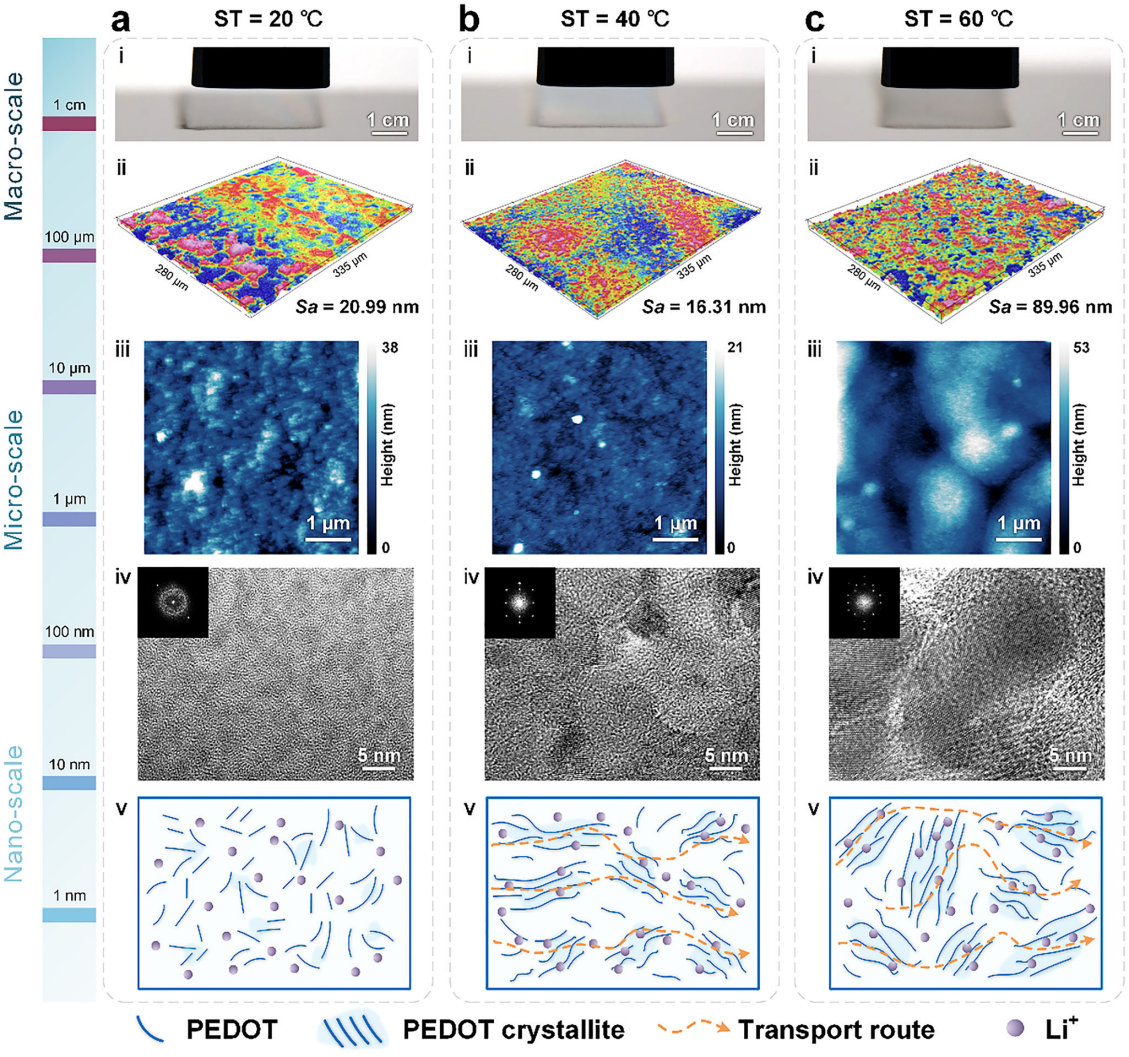
Multiscale structural evolution of WF‐AJP films under varying temperatures. a–c) PEDOT:PSS films printed at substrate temperatures of (a) 20  °C, (b) 40  °C, and (c) 60  °C. (i) Optical images of PEDOT:PSS films deposition (Macro‐scale). (ii) 3D surface topographies captured via white‐light interferometry (Micro‐scale). (iii) AFM scans showing micro‐scale surface morphology. (iv) TEM images with selected area electron diffraction (SAED) insets reveal PEDOT domain ordering (Nano‐scale). (v) Schematic diagrams illustrate PEDOT crystallite alignment and charge transport routes, moderate heating yields smoother surfaces and improved ordering.

To investigate the influence of substrate temperature on the multiscale morphology of printed PEDOT:PSS films, we systematically examined the surface topography (Table S4, Supporting Information) and defect formation at three representative temperature conditions. At 20 °C (**Figure** **4**a), the film exhibited moderate surface roughness (ii, *Sa* = 20.99 nm), with a relatively uniform appearance and no prominent defects on the macroscale (i). The aerosol droplets had sufficient time to spread before drying, leading to uniform deposition. Raising the substrate temperature to 40 °C (Figure 4b) yielded smoother surfaces (ii, *Sa* = 16.31 nm), attributed to moderately accelerated solvent evaporation that promotes uniform redistribution of PEDOT in the PSS matrix and suppresses local aggregation (v). The enhanced drying dynamics further contribute to improved molecular ordering, as evidenced by sharper nanoscale features and denser packing.

However, when the temperature was raised to 60 °C (Figure 4c), the surface roughness increased markedly (ii, *Sa* = 89.96 nm), accompanied by the formation of macrocracks at the film edges and microcracks near the center (Figure S9a, Supporting Information). This degradation results from excessive evaporation (iii), which disrupts the gradual reorganization of PEDOT domains and induces excessive segregation of insulating PSS.^[^ ^31^, ^32^ ^]^ Our observations are consistent with literature evidence that PEDOT:PSS films undergo temperature‐dependent microstructural reorganization and PSS redistribution.^[^ ^30^ ^]^ These effects reduce interparticle connectivity, hinder percolation pathways, and ultimately degrade conductivity.

Rapid solvent loss causes inhomogeneous shrinkage stress, particularly near the film edges, amplifying topographic variations and triggering mechanical failure (Figure S9b, Supporting Information). In the WF‐AJP process, aerosol deposition and in situ curing occur simultaneously, making the film quality highly sensitive to the thermal evaporation dynamics. An optimal temperature window is necessary to balance nanoscale ordering with macroscopic structural integrity. GIWAXS analysis reveals that the (010) π–π stacking peak of the PEDOT:PSS film shifts from *q* ≈ 1.78 Å^−1^ at 20 °C to *q* ≈ 1.80 Å^−1^ at 60  °C, indicating a reduced interchain spacing and enhanced molecular ordering at elevated substrate temperature during deposition (Figure S9c–e, Supporting Information). Notably, although elevated temperatures promote short‐range molecular ordering at the nanoscale, they concurrently exacerbate shrinkage‐induced cracking at the macroscale. These results highlight the need to balance nanoscale ordering and large‐area uniformity through precise thermal management, we identify 40 °C as the optimal processing window for uniform, crack‐free thin films.

### 2.4 Staggered Printing Strategy to Improve Large Area Film Uniformity

The uniformity of large‐area films is critical for ensuring the electrical, mechanical, and optical properties. However, during the layer‐by‐layer printing process, material distribution typically follows a Gaussian profile, leading to cumulative interlayer errors that result in surface undulations and uneven material accumulation, which affect the overall structural uniformity and functional stability of the film. Errors in the film manufacturing process primarily arise from two directions: in‐plane (within the same layer) and interlayer (across layers) directions. In the in‐plane direction, overlap of adjacent scan paths and uneven material distribution lead to local thickness deviations, forming periodic undulations. In the interlayer direction, material accumulation errors between layers compound with each successive layer.

**Figure 5:**
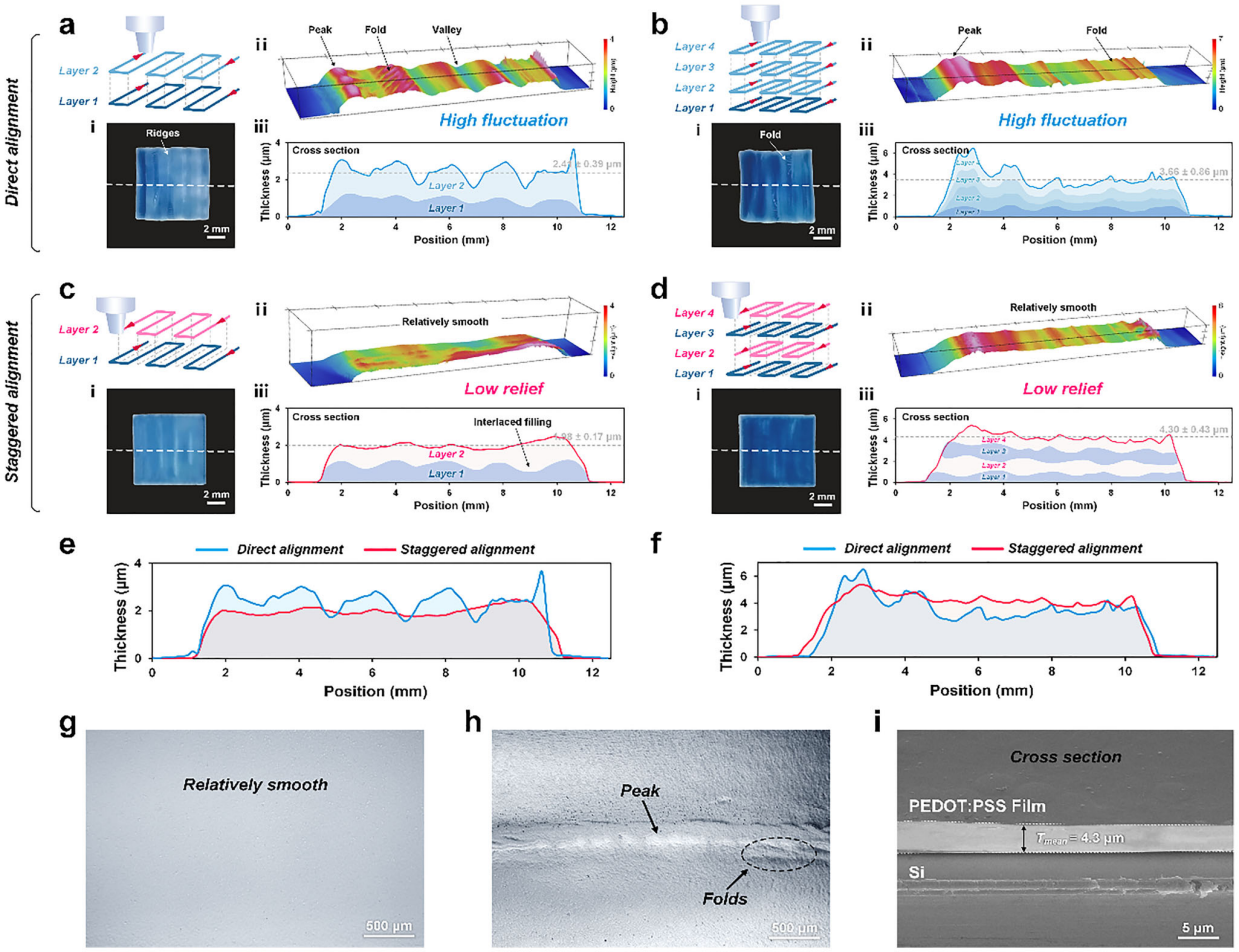
Staggered printing strategy in WF‐AJP. a,b) Direct alignment results in pronounced surface undulations, including peaks, folds, and valleys, as shown in the bilayer (a) and four‐layer (b) configurations. Subfigures show schematic diagrams (i), 3D surface topographies (ii), and cross‐sectional thickness profiles (iii). c,d) Staggered alignment leads to smoother surfaces with interlaced layer filling, reducing topographic relief, the bilayer (c) and four‐layer (d) films exhibit improved uniformity and minimized defects. e,f) Comparison of thickness profiles between direct and staggered alignment, staggered alignment significantly suppresses thickness fluctuation in both bilayer (e) and four‐layer (f) films.

Based on single‐line printing experiments, we determined the process parameters for WF‐AJP printing of films: *H* = 1 mm, SGFR = 300 sccm, CGFR = 600 sccm, *V* = 20 mm min^−1^, and the heating plate temperature set to 40 °C. Considering the Gaussian distribution characteristics, we set the scanning spacing to 2 mm, slightly smaller than the effective linewidth of the printed material, to reduce errors in the lateral direction and ensure that adjacent deposition paths uniformly cover the surface. We used direct alignment for double‐layer deposition and fabricated a square PEDOT:PSS film with dimensions of 1×1 cm^2^. As shown in **Figure** **5**a, the printed film appeared blue overall (i), with periodic band‐like color variations along the scanning direction. The edges of the film showed slight deformation. Darker blue regions indicate greater local film thickness. To further quantify the variation in film thickness, we conducted white‐light interferometric measurements along the white dashed line on the film. The cross‐sectional (iii) shows *T*_mean_ = 2.41 µm and *T*_std_ = 385 nm (16% of *T*_mean_). The thickness fluctuation range was 1.57 µm, corresponding to 65.2% of *T*_mean_, showing significant variation. In 3D morphology (ii), the peak and valley characteristics are clearly visible, corresponding to the periodic color variation in sample image (i). Additionally, folds were observed at the peaks, which are detrimental to the uniformity of the film. After the deposition of the first layer, the second layer continued to deposit on top of the existing morphology, causing the peaks and valleys to align directly, exacerbating the surface undulation (iii). The material accumulation at the peaks led to the accumulation of residual stress, ultimately resulting in folds at the peaks (Figure 5h) and deformation at the film edges.

To address uneven material deposition, we propose a staggered printing strategy, where the scan paths of the current layer are laterally offset relative to the previous layer to reduce the morphological unevenness caused by multilayer material accumulation. Since WF‐AJP in the direct alignment stacking mode forms distinct peak‐valley structures, we set the offset to half of the scanning spacing to achieve misaligned filling of peaks and valleys between adjacent layers (Movie S2, Supporting Information). As shown in Figure 5c, the scanning path of Layer 2 was offset by 1 mm without changing the scanning path of Layer 1. First, the color variation in the sample image became smoother, and the band‐like color distribution has weakened (i). Then, the 3D morphology is relatively flat (ii), with the peak‐valley structure almost disappearing, and no folds were observed. The cross‐sectional (iii) shows *T*_mean_ = 1.98 µm and *T*_std_ = 174 nm (8.8% of *T*_mean_). This represents a 44.9% decrease compared to the results from direct alignment deposition. The thickness fluctuation range was 0.76 µm, representing 38.4% of *T*_mean_, a 41.1% decrease from direct alignment deposition. These experimental results demonstrate that the staggered printing strategy significantly improves the uniformity of the printed film (Figure 5e; Figure S10a, Supporting Information). The peaks and valleys of Layer 1 and Layer 2 are staggered and filled, reducing interlayer cumulative errors and optimizing the morphology of the deposited film surface (Figure 5g) and edges. At film edges, the orientation‐dependent volumetric throughput of WF‐AJP was compensated by adjusting nozzle speed during swath‐to‐swath transitions.

To assess the applicability of the staggered printing strategy under higher deposition throughput and its impact on film uniformity, we increased the number of printed layers from 2 to 4, keeping the same printing parameters. As shown in Figure 5b, the film surface printed in direct alignment mode exhibits more pronounced band‐like color variations, with visible folds in the sample image (i). Cross‐sectional (iii) shows that the film has a mean thickness (*T*_mean_) of 3.67 µm and a standard deviation (*T*_std_) of 857 nm, representing 23.3% of *T*_mean_. A peak of 6.65 µm in height appeared near the end of the scanning path, resulting in a thickness fluctuation range of 4.17 µm, which is 113.6% of *T*_mean_. As the printing process is continuous, the cumulative effect of adding layers exacerbates interlayer material accumulation errors and limits the solvent evaporation rate. Microdroplets, under capillary forces, continue to accumulate toward the end of the printing path,^[^ ^33^ ^]^ ultimately forming a single‐sided peak due to deposition asymmetry (ii).

Figure 5d shows the deposition result for the staggered printing of 4 layers, where the film color is noticeably darker compared to the 2‐layer deposition, but it maintains good uniformity overall (i). The 3D morphology is relatively flat (ii), with small ripples present, and the film thickness near the end of the scanning path was slightly higher than on the other side. Cross‐sectional (iii) shows that the *T*_mean_ of film was 4.3 µm (Figure 5i), and the *T*_std_ was 433 nm, representing 10% of *T*_mean_, which is 57.1% lower than direct alignment deposition. The thickness fluctuation range is 2.22 µm, or 51.6% of *T*_mean_, which is much smaller than the 113.6% fluctuation seen in direct alignment deposition. However, compared to the 2‐layer staggered printing result, increasing the number of deposited layers leads to a slight decrease in uniformity (Figure 5f; Figure S10b, Supporting Information).

Overall, these results show that the staggered printing strategy eliminates the limitations imposed by the Gaussian distribution characteristics of single‐layer printing, effectively reducing interlayer material accumulation errors and improving overall film uniformity. The strategy shows good applicability under high‐throughput deposition.

### 2.5 Electrical Conductivity Characterization

**Figure 6:**
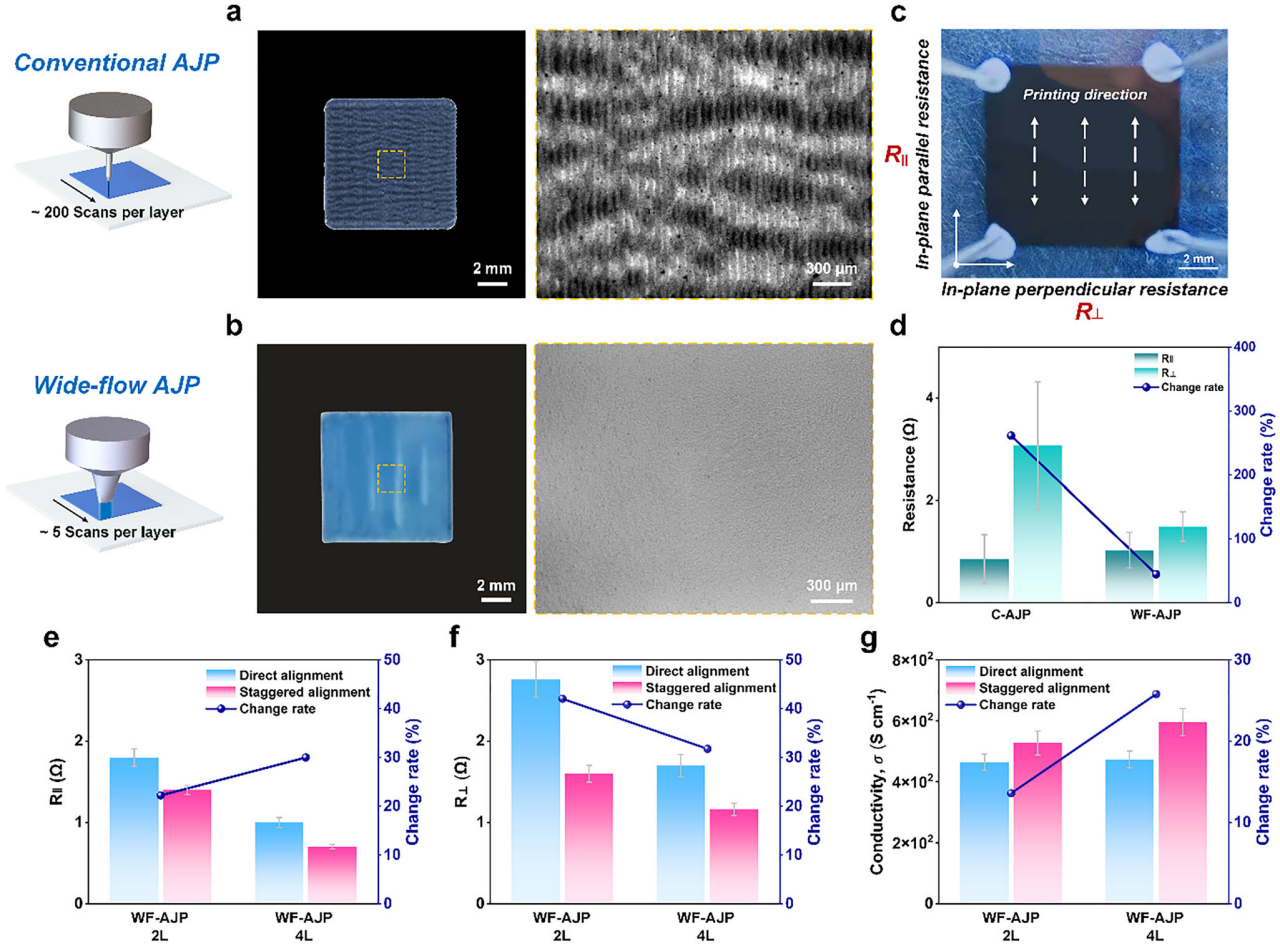
Electrical anisotropy regulation and conductivity enhancement of printed PEDOT:PSS films. a,b) Optical images of PEDOT:PSS films printed by conventional AJP (a) and WF‐AJP (b). c) Schematic diagram of in‐plane resistance measurement along parallel (*R*_∥_) and perpendicular (*R*_⊥_) directions relative to the printing direction. d) Comparison of *R*_∥_ and *R*_⊥_ values for conventional AJP and WF‐AJP films. e–g) Electrical performance of PEDOT:PSS films under different printing strategies and stacking layers: *R*_∥_ (e), *R*_⊥_ (f), and conductivity (g). Error bars represent standard deviation from *n* = 3 independent samples.

PEDOT:PSS films produced via conventional AJP typically exhibit significant in‐plane electrical anisotropy, with substantial resistance differences between the directions parallel and perpendicular to the printing path. This phenomenon results from the lateral stacking characteristics during printing, which enhances the continuity of conductive paths along the printing direction. In contrast, perpendicular conductive paths are affected by uneven particle accumulation and interface barriers, obstructing electron transport and significantly increasing resistance. PEDOT:PSS films were fabricated using conventional AJP (nozzle size: 300 µm) and compared with those made using WF‐AJP (**Figure** **6**a,b). Due to the narrow print width of conventional AJP, the 1×1 cm^2^ PEDOT:PSS films required 200 lateral scanning passes, forming a distinct linear stacking structure perpendicular to the scanning direction. Electrical measurements revealed a 261.18% resistance difference between the parallel (*R*_∥_ = 0.85 Ω ± 0.48 Ω) and perpendicular (*R*_⊥_ = 3.07 ± 1.25 Ω) directions, indicating that the nonuniform overlap regions between adjacent printed lines led to anisotropic charge transport and reduced overall conductivity.

In contrast, WF‐AJP improves in‐plane conductivity uniformity and overall electrical performance by increasing single‐pass scanning coverage, which effectively reduces the lateral deposition nonuniformity caused by multiple scanning layers (Figure 6b). Figure 6d compares the resistance differences between AJP and WF‐AJP under identical single‐layer scanning conditions. The results show that *R*_∥_ is similar for both methods (conventional AJP: 0.85 Ω, WF‐AJP: 1.03 Ω). However, the *R*_⊥_ decreases from 3.07 Ω (conventional AJP) to 1.49 Ω (WF‐AJP). This significant difference reflects the considerable improvement in *R*_⊥_ achieved with WF‐AJP. Although *R*_⊥_ for WF‐AJP remains higher than *R*_∥_, it is markedly reduced compared to conventional AJP. The wider deposition lines promote uniform solvent evaporation, enabling consistent PEDOT phase separation and forming a continuous conductive network that mitigates PSS‐induced barriers.

To assess the impact of the staggered scanning strategy on the electrical performance of PEDOT:PSS films, we compared WF‐AJP films printed with different scanning strategies (2‐layer stacking). As shown in Figure 6e–g, the direct alignment printed film exhibits *R*_∥_ = 1.8 Ω, *R*_⊥_ = 2.76 Ω, and conductivity (*σ*) = 540.7 S cm^−1^. In comparison, the staggered alignment printed film exhibited *R*_∥_ = 1.4 Ω, *R*_⊥_ = 1.6 Ω, and conductivity (*σ*) = 609.8 S cm^−1^, corresponding to a 12.8% increase in conductivity. This improvement is primarily attributed to the reduction in *R*_⊥_ achieved by staggered alignment, which promotes more uniform material deposition, reduces stress gradients, and prevents local stress concentration due to uneven solvent evaporation, thereby optimizing the conductive network in the perpendicular direction.

At higher deposition throughput, the benefits of staggered scanning become more pronounced. Figure 6e–g compares the electrical performance of films printed with direct and staggered alignment under higher scanning layers (4‐layer stacking). The direct alignment printed film exhibited *R*_∥_ = 1.0 Ω, *R*_⊥_ = 1.7 Ω, and conductivity (*σ*) = 494.1 S cm^−1^, while the staggered alignment printed film exhibited *R*_∥_ = 0.7 Ω, *R*_⊥_ = 1.2 Ω, and conductivity (*σ*) = 611.9 S cm^−1^, reflecting a 23.8% increase in conductivity. This improvement is more pronounced compared to the 2‐layer deposition. The enhancement is attributed to the aggregation of PEDOT particles at interfaces in direct alignment mode, which creates high‐resistance regions (as seen in the valley regions of Figure 5b), further limiting carrier transport in the thickness direction. In contrast, the staggered scanning strategy optimizes material deposition by allowing adjacent layers to fill misaligned areas, reducing interlayer resistance and improving contact quality, thereby enhancing electron transport in the thickness direction. This effect is amplified as the film thickness increases.

WF‐AJP demonstrates a significant advantage over conventional AJP in producing large‐area, uniform films. The staggered scanning strategy further optimizes the microstructure of PEDOT:PSS films, improving in‐plane conductivity uniformity and reducing resistance in the thickness direction (Figure S11, Supporting Information). This approach offers a promising optimization strategy for high‐performance PEDOT:PSS film manufacturing, with significant potential for applications in flexible electronics, transparent conductive films, and large‐area printed electronic devices.

### 2.6 Process Efficiency and Performance Enhancement

**Figure 7:**
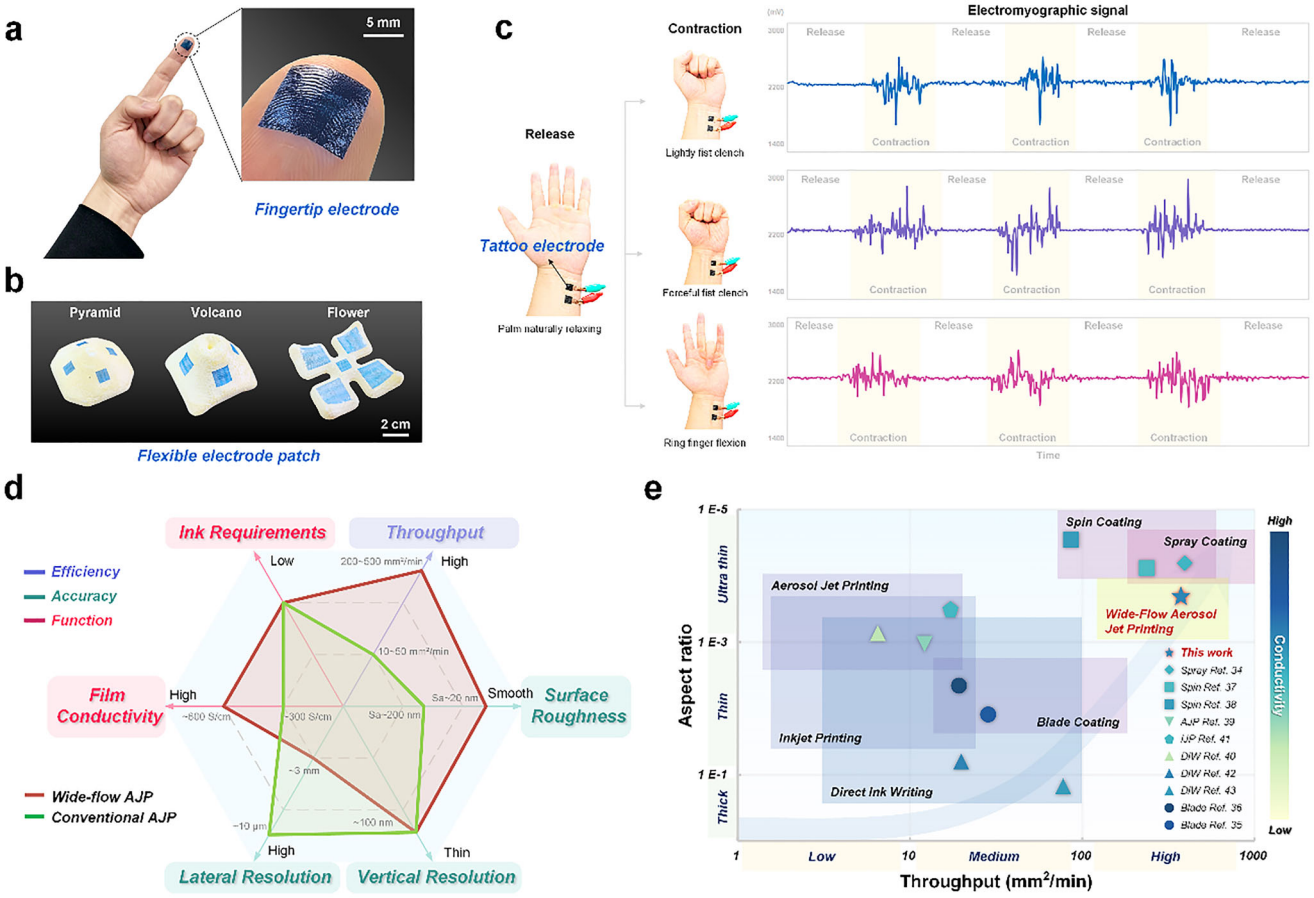
Application potential and technical advantages of WF‐AJP. a) PEDOT:PSS film (1×1 cm^2^) deposited via WF‐AJP conformally attached to a fingertip, showing strong adhesion and microstructural interlocking. b) WF‐AJP films applied to complex 3D surfaces, including volcano, pyramid, and flower geometries, confirming multiscale adaptability. c) Tattoo electrodes (1×1 cm^2^) laminated on the wrist for EMG signal acquisition during various hand motions. d) Radar plot comparing WF‐AJP and conventional AJP in throughput, roughness, resolution, conductivity, and ink compatibility (*n* ≥ 3). e) Comparative plot of printing throughput versus electrical conductivity for various PEDOT:PSS‐based thin film deposition techniques.

To evaluate the versatility and practical application potential of WF‐AJP technology, we designed and implemented three proof‐of‐concept application cases. Compared with spray deposition, which primarily produces uniform coatings, WF‐AJP offers spatial selectivity and digital patterning capability, thereby expanding its applicability to functional device integration. First, we conformed a 1×1 cm^2^ PEDOT:PSS film onto the surface of a fingertip as an epidermal electrode. As shown in **Figure** **7**a, the PEDOT:PSS film demonstrated excellent adhesion and structural conformability, tightly fitting the fingerprint texture on the fingertip and forming a micro‐scale interlocking interface. Next, we attached films of various sizes as flexible electrode patches to typical nonplanar structures, including volcano‐shaped, pyramid‐shaped, and flower‐shaped models (Figure 7b). 1 × 1 cm^2^ films were conformally attached around the pyramid, 1.5 × 1.5 cm^2^ films around the volcano, 2 × 2 cm^2^ films on the petals, and a 1 × 1 cm^2^ film at the flower center. The results showed that the films printed using WF‐AJP adhered well to complex geometries with varying scales and curvatures, demonstrating excellent conformability. Finally, as shown in Figure 7c, we placed a pair of 1 × 1 cm^2^ PEDOT:PSS films (thickness ≈5 µm, prepared by 4‐layer stacking) on the wrist as tattoo electrodes (Figure S12, Supporting Information) for electromyography (EMG). Due to the ultra‐thin thickness and negligible weight of the electrodes, they did not significantly impede the wearer's movement, and the skin at the attachment site could deform freely and reversibly. EMG signals were collected during three muscle activation states: light fist clenching, strong fist clenching, and ring finger flexion, with each action repeated three times (Movie S3, Supporting Information). The signal results showed that the muscle responses from different actions were distinguishable and consistent, confirming the potential of the WF‐AJP constructed tattoo electrodes for applications in bioelectrical monitoring, human‐computer interaction, and wearable electronics.

As a novel functional film fabrication process, we systematically evaluated the advantages of WF‐AJP compared to conventional AJP in terms of efficiency, precision, and functionality (Figure 7d). The parameters included printing throughput, film surface roughness, vertical resolution, lateral resolution, film conductivity, and ink material compatibility. WF‐AJP achieved a significant improvement in printing throughput compared to conventional AJP while maintaining excellent conductivity and surface smoothness. Additionally, the vertical resolution of WF‐AJP can be controlled at the submicron level, with the lateral printing width extending to millimeter level, resulting in an aspect ratio of ≈1:6000, making it suitable for constructing wide, low‐profile structures. More importantly, these performance improvements were achieved without sacrificing ink material compatibility, showcasing its enormous potential in scalable, patterned functional film manufacturing.

To further assess the process advantages of WF‐AJP in functional film fabrication, we conducted a comparative analysis (Figure 7e), evaluating representative PEDOT:PSS‐based printing approaches with secondary dopants. The results indicate that conventional high‐throughput techniques^[^ ^34^, ^35^, ^36^, ^37^, ^38^ ^]^ suffer from limited patterning capability, whereas high‐precision methods^[^ ^39^, ^40^, ^41^, ^42^, ^43^ ^]^ are constrained by scalability and diminished functional performance. WF‐AJP effectively bridges this gap by achieving patterned deposition of ultra‐low aspect ratio films (< 1:5000) with a measured thickness of 1.98 ± 0.17 µm, while maintaining high throughput (365.1 mm^2^ min^−1^) and high conductivity (*σ* > 600 S cm^−1^), providing a promising route for scalable, pattern‐defined functional film manufacturing.

## 3 Conclusion

In conclusion, this work presents a universal and high‐throughput strategy termed WF‐AJP for the scalable fabrication of ultra‐low aspect ratio functional films. By reshaping the nozzle into a flattened rectangular geometry, the system generates planar aerosol jets that enable millimeter‐scale width deposition in a single pass (aspect ratio < 1:6000, thickness < 500 nm). The evolution of the film formation process was systematically investigated through cross‐scale characterization, revealing a balance between macroscopic morphology and functional performance. To further enhance film uniformity and suppress interlayer accumulation, a staggered deposition strategy was employed, resulting in highly uniform micron‐thick PEDOT:PSS films (aspect ratio < 1:5000, thickness < 2 µm, *Sa* = 27.9 nm). Demonstrations, including conformal fingertip electrodes and tattoo‐like sensors, validated the platform's suitability for flexible electronics and bio‐integrated devices, highlighting its scalability, structural precision, and surface adaptability. The integration of precise structural control with scalable, high‐throughput patterning opens new avenues for advanced functional film development.

## 4 Experimental Section

### WF‐AJP System

WF‐AJP system was custom‐designed, as depicted in Figure S4 (Supporting Information). It consists of a process control module, a vision module, and a printing module. The process control module includes a three‐axis motion platform (Guruitech, China), two gas flow controllers (Jednl Automation, China), and an industrial control unit, enabling precise regulation of deposition parameters. A monitoring camera (Hikrobot, China) was used for real‐time tracking and feedback to ensure accurate alignment during printing. The printing module integrates an ultrasonic atomizer and a WF‐AJP nozzle, allowing for controlled material deposition. A heated substrate stage was integrated to facilitate in‐situ thermal processing, enhancing solvent evaporation and improving film adhesion. The WF‐AJP nozzle was designed as a monolithic structure, integrating the flow channel and nozzle outlet to ensure stable and uniform aerosol delivery. Compared with conventional split‐type designs, the integrated WF‐AJP nozzle further optimizes flow continuity and structural stability.^[^ ^44^, ^45^ ^]^ The nozzle outlet features a rounded‐rectangular geometry (4×1 cm^2^), optimized to minimize turbulence and pressure losses during deposition (Figure S2, Supporting Information). The entire nozzle structure was fabricated using UV‐curable 3D printing, followed by ultrasonic cleaning in acetone and isopropanol to eliminate residual polymer residues. To further eliminate contaminants and ensure the integrity of the internal structure, compressed nitrogen purging was employed, effectively preventing potential performance deviations due to structural imperfections.

### Ink and Substrate Preparation

Conductive structures, including lines and films, were printed via conventional and WF‐AJP systems. An aqueous dispersion of Poly(3,4‐ethylenedioxythiophene):polystyrene sulfonate (PEDOT:PSS, Clevios™ PH1000, Heraeus) was used as the base ink. To enhance the printability and electrical performance, PEDOT:PSS inks were modified with secondary dopants. Detailed formulation procedures are provided in the Supplementary Information, and the corresponding material properties are summarized in Table S1 (Supporting Information). Silicon wafers and float glass substrates (5×5 cm^2^) were employed for thin film deposition. To remove organic residues and particulate contaminants, the substrates underwent a sequential ultrasonic bath in acetone, isopropanol, and deionized water, with each step lasting 10 min. Following cleaning, the substrates were dried under a nitrogen stream and treated with oxygen plasma (100 W, 15 min) to enhance surface wettability and improve adhesion between the PEDOT:PSS ink and the substrates.

### Particle Motion in Coupled Multiphysical Field

To simulate the droplet deposition behavior during the WF‐AJP process, a coupled multiphysics model based on computational fluid dynamics (CFD) was developed (Figure S1, Supporting Information). The model integrates the continuity, momentum, and energy conservation equations, incorporating a discrete phase model (DPM) to inject atomized microdroplets and track the transport trajectories of aerosol particles within the high‐velocity carrier gas flow. Upon particle impingement on the substrate, the Eulerian wall film (EWF) model is employed to capture the transformation between discrete particles and wall‐bound liquid films, enabling the numerical description of particle adhesion and film formation at solid boundaries. Under the incompressible flow assumption, the volume of fluid (VOF) method was used to resolve the evolution of the droplet interface morphology during deposition. Coupling between the VOF and EWF models allows the interaction between the continuous fluid phase and the wall film to be accurately captured. Additionally, when atomized droplets impact existing liquid surfaces, the DPM‐VOF model enables dynamic phase conversion from the particle phase to the fluid domain, thereby capturing the multiscale interfacial coupling and flow dynamics during high‐throughput printing.

### Fabrication of PEDOT:PSS Films

WF‐AJP system was operated via a graphical user interface (GUI), controlling the x‐, y‐, and z‐axis motion platforms to ensure precise deposition. A vision monitoring system (Hikrobot, China) was integrated with the printing nozzle, enabling real‐time observation of aerosol jet dynamics and deposition behavior. The carrier gas flow rate (CGFR), sheath gas flow rate (SGFR), and ink atomization rate were dynamically adjusted to regulate aerosol stream width and deposition consistency, ensuring stable and uniform thin film formation. Each PEDOT:PSS thin film unit (1×1 cm^2^) was printed with a path spacing equal to the effective trace width of the deposited material, minimizing overspray and improving film uniformity. Due to the sub‐micron layer thickness, the z‐height remained fixed during multilayer deposition, preventing cumulative positioning errors and ensuring consistent film morphology. To further enhance film uniformity and electrical properties, an in‐situ thermal treatment was applied via a substrate heating stage maintained within a 20–60 °C temperature range. This real‐time heating accelerated solvent evaporation, improved film‐substrate adhesion, and reduced residual moisture, optimizing film conductivity.

### Characterization and Measurement

Effective trace width and overspray width of the WF‐AJP deposition were measured using an optical microscope (Cossim, China), based on a consistent measurement protocol. Along the printing direction, the maximum deposition width *L*_max_ was extracted on each pixel column in orthogonal directions. Next, the discontinuous pixel units along the printing direction were removed, and a continuous deposition width *L*_i_ along the printing direction was obtained. Finally, the *LW* of the printed traces was denoted as Equation (4). The maximum printing range was defined as Equation (5). The width of overspray was denoted as Equation (6).^[^ ^25^ ^]^

(4) $$LW=\frac{1}{2}\sum L_{i}$$

(5) $$PW=\frac{1}{n}\sum L_{\max}$$

(6) $$OW=\frac{1}{2}\left(PW-LW\right)$$

Microstructure and surface morphology were characterized via field emission scanning electron microscopy (Hitachi, Japan) at an accelerating voltage of 5 kV and a working distance of 10 mm, with both secondary electron and backscattered electron modes employed. Representative SEM images were used to reveal the morphology at the microscale. Atomic force microscopy (Asylum Research, USA) was employed in tapping mode to characterize the nanoscale morphology and surface roughness of the printed PEDOT:PSS films. Scan areas of 1×1 µm2 and 5×5 µm2 (collected from multiple independent regions, *n* = 3) were analyzed to assess topographical uniformity across different length scales. Transmission electron microscopy (JEOL, Japan) was utilized to investigate the internal microstructure and crystallinity of the films. PEDOT:PSS ink was directly printed onto carbon‐coated copper grids (300 mesh) via WF‐AJP and dried to form films. Imaging was conducted at an accelerating voltage of 200 kV, and fast Fourier transform analysis was performed to evaluate lattice ordering and π–π stacking characteristics.

Surface roughness and topographical features were analyzed using a 3D confocal white light interferometer (Sensofar, Spain) in noncontact mode, with a lateral resolution of 0.1 µm and a vertical resolution of 10 nm. The average surface roughness (*Sa*) and maximum peak‐to‐valley height (*Sz*) were extracted from three scanned areas per sample. In order to evaluate the structural characteristics of the film samples, the average film thickness was defined as Equation (7), the root mean square film thickness was defined as Equation (8), the standard deviation of film thickness was defined as Equation (9), and the thickness fluctuation range was defined as Equation (10).

(7)

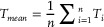



(8)

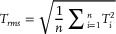



(9)

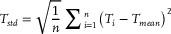

(10) $$T_{range}=T_{\max}-T_{\min}$$

GIWAXS measurements were performed to investigate the molecular packing of the PEDOT:PSS films. 2D scattering patterns were recorded and integrated to obtain out‐of‐plane line‐cut profiles, from which the (010) π–π stacking peak position was analyzed.

Electrical conductivity was assessed by measuring the sheet resistance using a four‐point probe system (Keithley, USA), ensuring reproducibility through multiple measurements at different locations on each sample.

### Statistical Analysis

Unless otherwise specified, all data points plotted indicate the mean ± standard deviation (SD), with *n* = 3 independently printed samples per group. Conductivity values (Figure S6, Supporting Information) were obtained from four‐point probe measurements of sheet resistance at 3 positions per sample, combined with the average film thickness measured at 6 regions. The data points and ranges in the technique comparison (Figure 7d,e) are derived either from the experimental measurements (*n* ≥ 3) or compiled from previously reported literature values. Statistical analysis and plotting were performed using Origin, Version 2022 (OriginLab Corporation, Northampton, MA, USA).

### Ethical Declaration

The functional demonstration (Figure 7; Figure S12 and Movie S3, Supporting Information) involved noninvasive testing of a wearable patch on the skin surface for demonstration purposes, with no risk or discomfort to the participant. All participants provided informed consent. According to institutional guidelines, formal ethics committee approval was not required for this nonclinical study.

## Conflict of Interest

The authors declare no conflict of interest.

## Supporting information



Supporting Information

Supplemental Movie 1

Supplemental Movie 2

Supplemental Movie 3

## Data Availability

The data that support the findings of this study are available from the corresponding author upon reasonable request.
