# Supplementary material for: Wide‐Flow Aerosol Jet Printing Enables High‐Throughput, Ultra‐Low Aspect Ratio Patterning
Source: Adv Sci (Weinh). 2025 Nov 3;13(3):e12557. doi: 10.1002/advs.202512557 (PMC12806333; doi:10.1002/advs.202512557)
Supplement: Supplementary file 1 — Supporting Information [file ADVS-13-e12557-s002.docx]

**Supporting Information**

**Wide-Flow Aerosol Jet Printing Enables High-Throughput, Ultra-Low Aspect Ratio Patterning**

*Zenan Niu^1^, Hao Yi^1^*, Yufeng Jin^1^, Yaru Yue^2^, Shanshan Chen^2^*, Zhixu Dong^3^, Jia An^4^, Chee Kai Chua^4,5^, Huajun Cao^1^*

^1^State Key Laboratory of Mechanical Transmission for Advanced Equipment, Chongqing University, Chongqing, 400044, China.

^2^MOE Key Laboratory of Low-Grade Energy Utilization Technologies and Systems, School of Energy & Power Engineering, Chongqing University, Chongqing 400044, China.

^3^School of Mechanical Engineering, Shenyang University of Technology, Shenyang 110870, China.

^4^Centre for Healthcare Education, Entrepreneurship and Research @ SUTD (CHEERS), Singapore University of Technology and Design, 8 Somapah Road, Singapore 487372, Singapore.

^5^Department of Mechanical Engineering, Wuhan University of Science and Technology, Wuhan, 430081, China

*Corresponding authors Email: haoyi@cqu.edu.cn (H. Yi); shanshanchen@cqu.edu.cn (S. Chen)


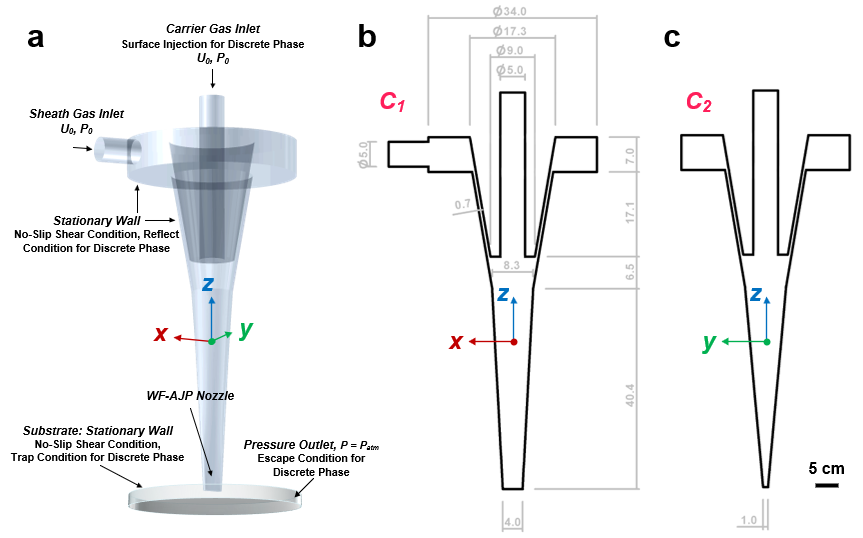


Figure S1. Representation of the WF-AJP CFD model. (a) boundaries and their corresponding boundary conditions, and (b) various key dimensions of the print channel.

Table S1. Summary of material properties.

| **Domain** | **Parameter** | **Symbol** | **Values** |
| --- | --- | --- | --- |
| Ink  (PEDOT:PSS) | Viscosity of ink | *μ* | 39.49 *mPa·s* |
|  | Surface tension of ink | *σ* | 0.07181 *N/m* |
|  | Density of ink | *ρ_i_* | 1050 *kg/m^3^* |
|  | Contact angle | *α* | 53.67° |
| Air | Density of air | *ρ_a_* | 1.225 *kg/m^3^* |
|  | Viscosity of air | *μ_a_* | 1.7894e-05 *kg/(m·s)* |

**Ink preparation**

PEDOT:PSS-secondary dopants inks were prepared by incorporating lithium bis (TFSI) into an aqueous dispersion of PH1000 (Clevios™ PH1000, Heraeus). The mixture was vigorously stirred using a magnetic stirrer for 30 minutes to yield a homogeneous solution with a final concentration of 45 wt%. To ensure optimal printability and improve dispersion stability, the resulting ink was further homogenized by magnetic stirring at 500 rpm for an additional 30 minutes at room temperature prior to deposition.


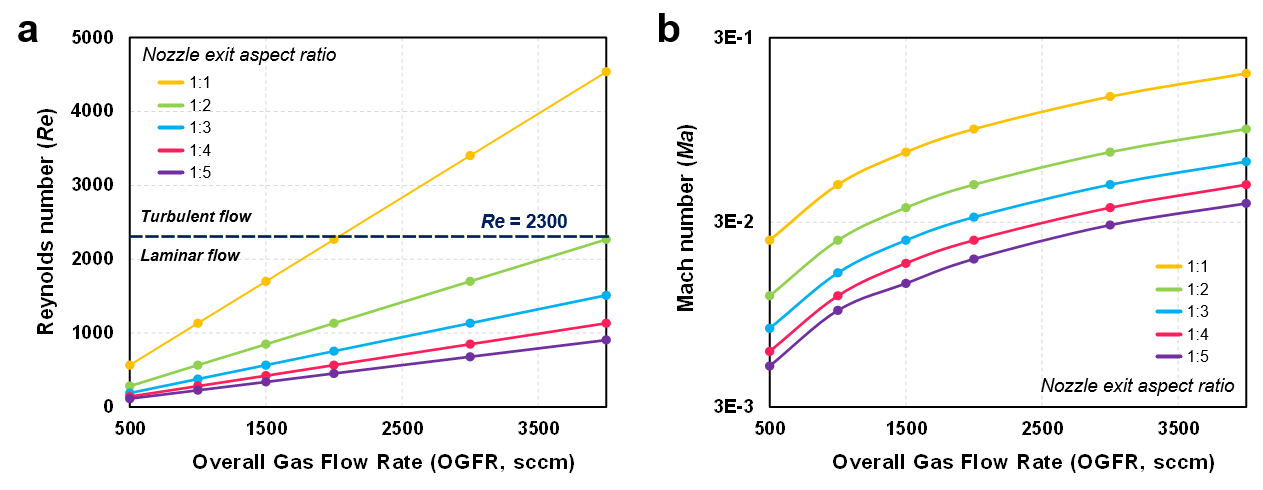


Figure S2. Fluid dynamics analysis of wide-flow aerosol jet printing under varying nozzle aspect ratios. (a) Reynolds number (*Re*) as a function of overall gas flow rate (OGFR) for nozzles with different exit aspect ratios (1:1 to 1:5). The dashed line indicates the laminar-to-turbulent transition threshold (*Re* = 2300). Lower aspect ratio nozzles maintain laminar flow across a wider range of flow rates. (b) Mach number (*Ma*) versus OGFR under varying nozzle aspect ratios. Higher aspect ratio nozzles reduce the compressibility effect by maintaining lower Ma values at a given flow rate.

$Re=\frac{\rho UL}{\mu}$ (S1)

where 𝜌 is the fluid density, 𝑈 is the average flow velocity, 𝐿 is the nozzle characteristic length, and 𝜇 is the dynamic viscosity of the fluid.

$Ma=\frac{U}{\sqrt{\gamma RT}}$ (S2)

where 𝑈 is the flow velocity, 𝛾 is the specific heat ratio, 𝑅 is the specific gas constant, and 𝑇 is the absolute temperature.

Based on the operating conditions and nozzle geometry, the Reynolds and Mach numbers were computed to assess the flow regime and compressibility. The results showed that the flow remained laminar (*Re* < 2300) and subsonic (*Ma* < 0.3) across all tested flow rates, ensuring flow stability and minimal compressibility effects.


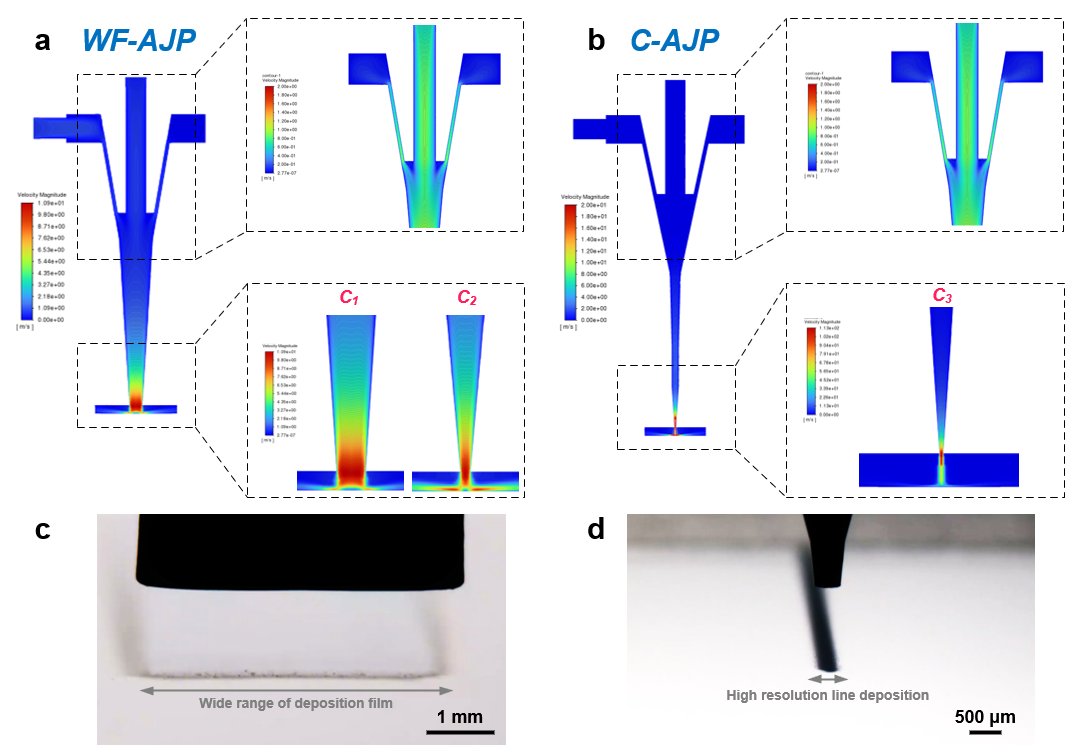


Figure S3. Comparison of flow dynamics and jet morphology between WF-AJP and C-AJP. (a and b) Simulated gas velocity profiles of the nozzle interior for (a) WF-AJP (SGFR = 600 sccm, CGFR = 600 sccm) and (b) C-AJP (SGFR = 80 sccm, CGFR = 100 sccm). Insets show the flow field cross-sections along the vertical axis and at the outlet. For WF-AJP, two orthogonal cross-sections are extracted: the long-axis section (*C₁*) and the short-axis section (*C₂*), showing anisotropic velocity gradients. For C-AJP, a single circular cross-section (*C₃*) represents symmetric flow behavior. (c and d) Optical images of the aerosol stream exiting the nozzle for (c) WF-AJP (nozzle size: 4×1 mm) and (d) C-AJP (nozzle size: 300 μm), revealing the stable and expanded planar jet in WF-AJP and the focused, narrow stream characteristic of C-AJP.


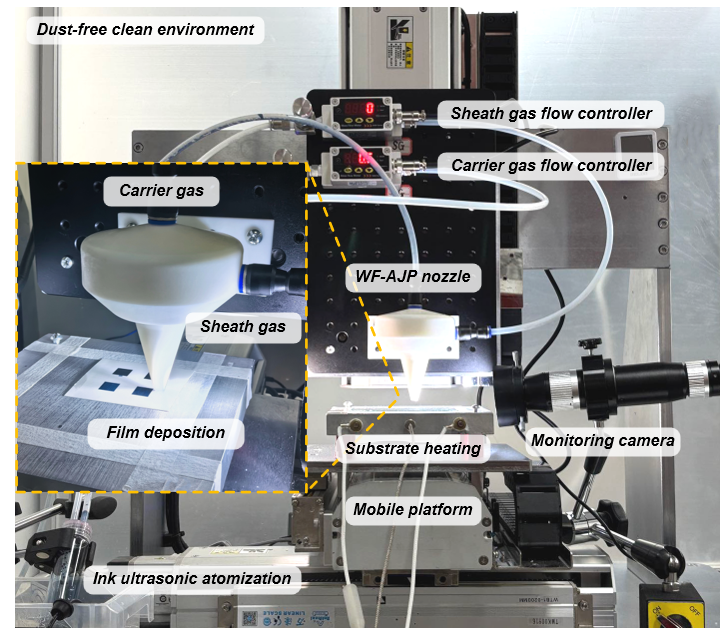


Figure S4. Photograph of the custom-built WF-AJP system. The system integrates three-axis motion platform (Guruitech, China), gas flow controller (Jednl Automation, China), industrial control unit, monitoring camera (Hikrobot, China), ultrasonic atomizer, heated substrate stage and WF-AJP nozzle.

**Software**

The 3D model of the print nozzle was designed using SolidWorks. Physical field modeling and particle motion simulation were performed using ANSYS Fluent (Under a licensed academic version) to analyze aerosol jet dynamics. MVS software was used for real-time data acquisition from an industrial matrix camera, enabling precise monitoring of the deposition process. AutoCAD was employed for print path planning, ensuring accurate trajectory control during patterning. SensoVIEW was utilized for generating topographic cloud representations of the printed samples, facilitating detailed surface analysis.


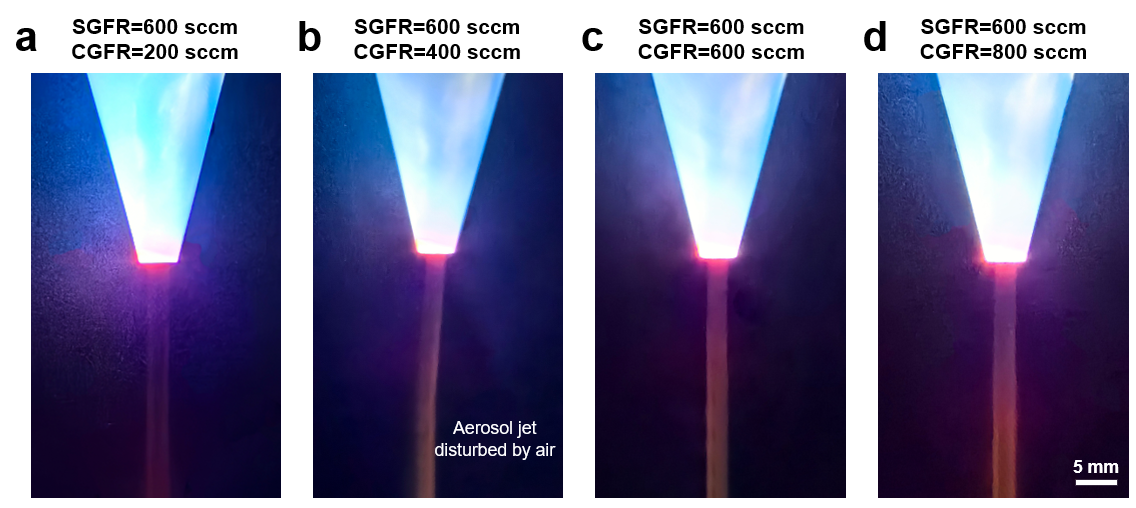


Figure S5. Visualization of WF-AJP behavior under varying carrier gas flow rate at a constant sheath gas flow rate. (a to d) show the evolution of the aerosol stream as CGFR increases from 200 sccm to 800 sccm. At low CGFR (a), the aerosol jet is weak and highly divergent. Moderate CGFR (b) leads to instability and disturbance by ambient air. As CGFR increases (c and d), the aerosol jet becomes progressively more collimated and stable, indicating improved focusing and printing suitability.

Table S2. Deposition thickness of printed line at different printing speeds.

| **Printing speed (mm/min)** | **SGFR (sccm)** | **CGFR (sccm)** | **H (mm)** | **Thickness (****μm)** |
| --- | --- | --- | --- | --- |
| 10 | 600 | 600 | 1 | 2.36 |
|  | 600 | 600 | 1 | 1.61 |
|  | 600 | 600 | 1 | 1.78 |
| 20 | 600 | 600 | 1 | 1.38 |
|  | 600 | 600 | 1 | 1.08 |
|  | 600 | 600 | 1 | 0.78 |
| 30 | 600 | 600 | 1 | 0.81 |
|  | 600 | 600 | 1 | 0.75 |
|  | 600 | 600 | 1 | 1.17 |
| 50 | 600 | 600 | 1 | 0.69 |
|  | 600 | 600 | 1 | 0.2 |
|  | 600 | 600 | 1 | 0.52 |
| 90 | 600 | 600 | 1 | 0.19 |
|  | 600 | 600 | 1 | 0.2 |
|  | 600 | 600 | 1 | 0.45 |


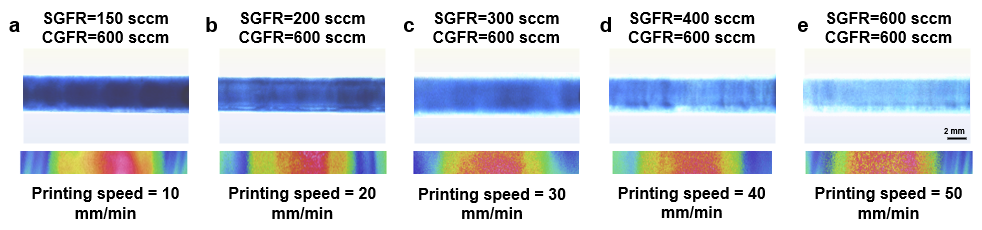


Figure S6. Influence of printing speed on deposition thickness in WF-AJP. (a to e) Optical micrographs (top) and white light interferometry profiles (bottom) of PEDOT:PSS films printed at varying speeds from 10 to 50 mm/min under fixed CGFR = 600 sccm. Film thickness shows significant variation with printing speed. Each white light interferometry scan captures a cross-sectional view along the width of the printed line.

Table S3. Effect of focus ratio on deposition line width and thickness.

| **Focus ratio** | **SGFR (sccm)** | **CGFR (sccm)** | **Printing speed (mm/min)** | **H (mm)** | **Line** **width (mm)** | **Thickness (μm)** |
| --- | --- | --- | --- | --- | --- | --- |
| 0.25 | 150 | 600 | 20 | 1 | 3.83 | 0.33 |
|  | 150 | 600 | 20 | 1 | 4.05 | 0.65 |
|  | 150 | 600 | 20 | 1 | 3.88 | 0.43 |
| 0.33 | 200 | 600 | 20 | 1 | 3.31 | 0.63 |
|  | 200 | 600 | 20 | 1 | 3.44 | 0.96 |
|  | 200 | 600 | 20 | 1 | 3.66 | 1.11 |
| 0.50 | 300 | 600 | 20 | 1 | 3.15 | 1.42 |
|  | 300 | 600 | 20 | 1 | 3.42 | 1.59 |
|  | 300 | 600 | 20 | 1 | 2.97 | 1.25 |
| 0.66 | 400 | 600 | 20 | 1 | 3.1 | 1.92 |
|  | 400 | 600 | 20 | 1 | 2.96 | 2.08 |
|  | 400 | 600 | 20 | 1 | 3.24 | 1.76 |
| 1.00 | 600 | 600 | 20 | 1 | 2.52 | 2.21 |
|  | 600 | 600 | 20 | 1 | 2.85 | 2.41 |
|  | 600 | 600 | 20 | 1 | 2.97 | 2.22 |


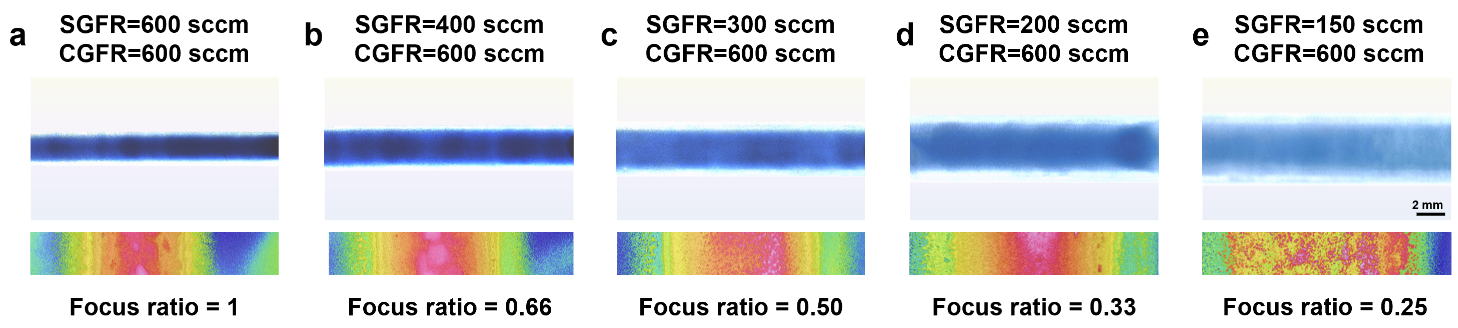


Figure S7. Geometric features evolution of printed films under different focus ratios in WF-AJP. (a to e) Optical images (top) and corresponding white light interferometry profiles (bottom) of PEDOT:PSS films printed with a fixed CGFR of 600 sccm and varying SGFR to adjust the focus ratio. As the focus ratio increases from 0.25 to 1.0, the width and thickness of the printed films change significantly.


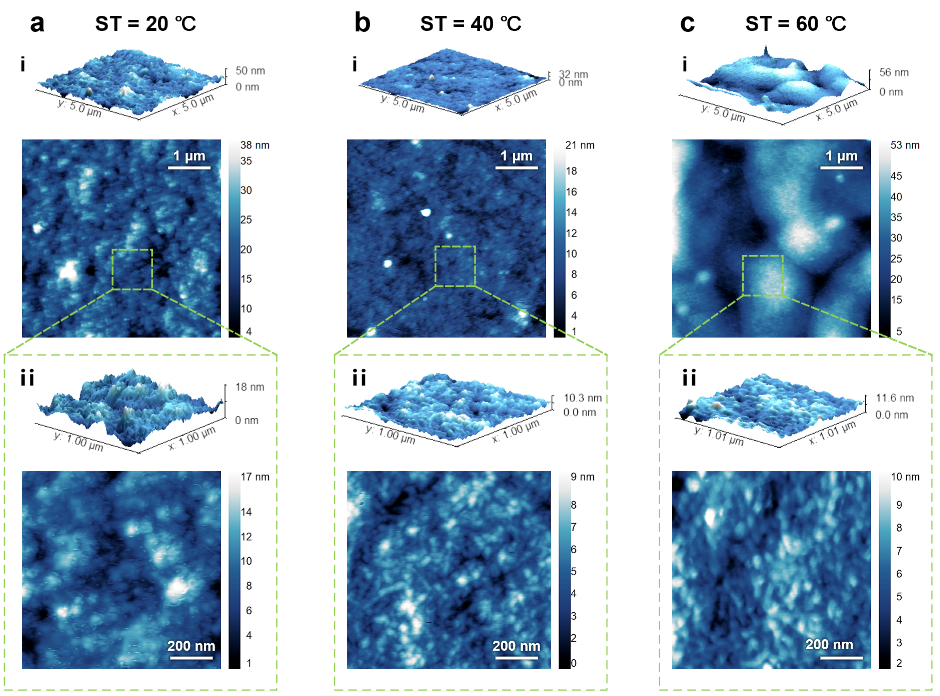


Figure S8. Temperature-dependent evolution of surface morphology in WF-AJP films. (a to c) PEDOT:PSS films printed at substrate temperatures of 20 °C (a), 40 °C (b), and 60 °C (c). (i) 5×5 μm AFM scans and corresponding 3D height maps show differences in surface texture and vertical roughness. (ii) Magnified 1×1 μm regions with corresponding 3D views reveal nanoscale granularity and height fluctuations. Optimal substrate heating at 40 °C yields a more uniform surface with lower roughness, while elevated temperature (60 °C) results in larger granular domains and increased height variation, indicating morphological instability.


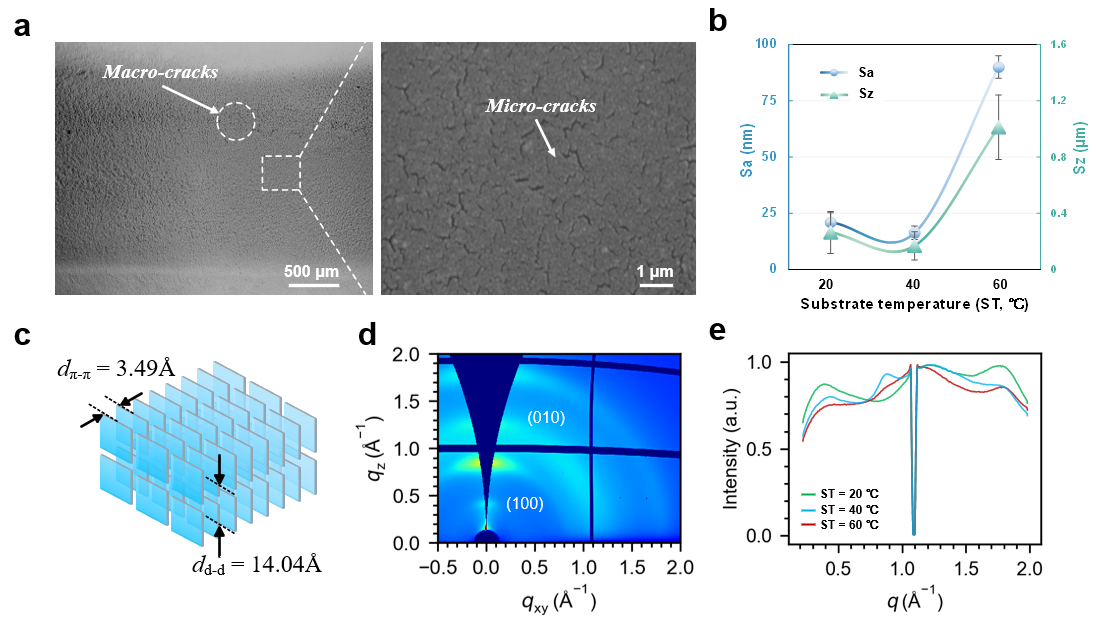


Figure S9. Temperature-dependent microstructure of WF-AJP films. (a) SEM images of PEDOT:PSS films at 60 °C reveal both macro-cracks and micro-cracks, indicating excessive thermal stress and non-uniform drying at elevated temperature. (b) Quantitative evolution of surface roughness parameters (*Sa* and *Sz*) as a function of substrate temperature. (c) Schematic illustration of the molecular packing structure in PEDOT:PSS thin films (40°C). (d) 2D GIWAXS pattern of a representative film (40°C). (e) One-dimensional *I*–*q* profiles extracted from GIWAXS data under different substrate temperatures (*ST* = 20 °C, 40 °C, and 60 °C), demonstrating the influence of substrate temperature on the degree of molecular ordering and crystallinity.

Table S4. Effect of substrate temperature on surface topography (ISO 25178).

| **T (℃)** | **Sa (nm)** | **Sq (nm)** | **Sz (μm)** | **Sdc* (nm)** | **Smc* (nm)** | **Smr* (%)** |
| --- | --- | --- | --- | --- | --- | --- |
| 20 | 20.99 | 29.69 | 0.26 | 78.58 | 30.18 | 47.90 |
| 40 | 16.31 | 20.95 | 0.17 | 50.45 | 27.83 | 45.13 |
| 60 | 89.96 | 114.62 | 1.01 | 249.70 | 155.20 | 43.94 |

**Note:** Values represent the average of *n* = 3 independent scan areas per condition.


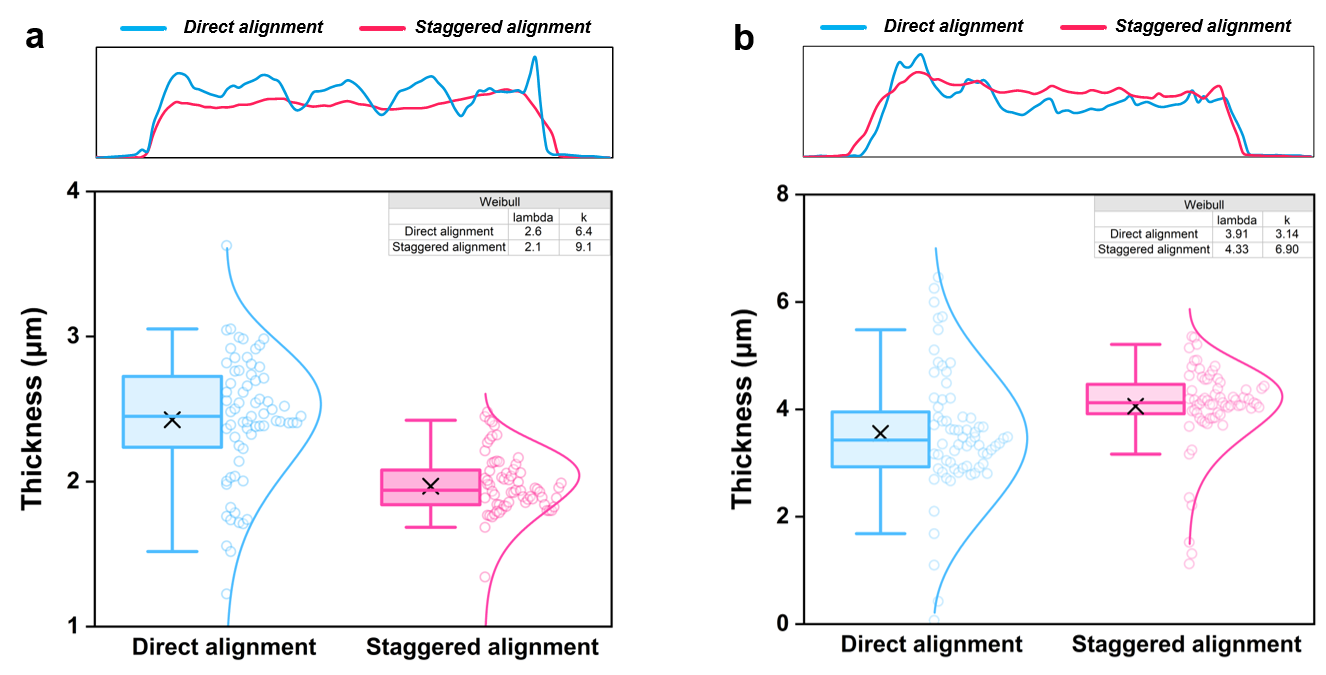


Figure S10. Effect of staggered alignment on thickness uniformity of WF-AJP films. (a and b) Thickness profiles (top) and box plots (bottom) comparing direct and staggered alignment strategies under both bilayer (a) and four-layer (b) WF-AJP deposition. The thickness distribution is more concentrated under staggered alignment, with reduced spread and smoother profiles. Weibull fitting parameters (insets) indicate a narrower distribution (*k*) and slightly lower or comparable scale parameter (*λ*), confirming enhanced uniformity and consistency. The statistical analysis confirms the effectiveness of the staggered strategy in suppressing interlayer accumulation and mitigating edge buildup.

Table S5. Electrical anisotropy and film properties of PEDOT:PSS films printed by conventional and wide-flow AJP.

| **Preparation method** | |  | ***R*_∥_ (Ω)** | ***R*_⊥_ (Ω)** | ***Rr* (Ω)** | ***F*** | ***Rs* (Ω)** | **Thickness (nm)** | ***σ* (S/cm)** |
| --- | --- | --- | --- | --- | --- | --- | --- | --- | --- |
| **C-AJP** |  | #1 | 0.60 | 2.20 | 3.67 | ***NA*** | ***NA*** | 3627 | ***NA*** |
|  |  | #2 | 0.54 | 2.50 | 4.63 | ***NA*** | ***NA*** | 3731 | ***NA*** |
| **WF-AJP 2L** | Direct alignment | #3 | 1.80 | 2.76 | 1.53 | 0.95 | 9.33 | 1983 | 540.72 |
|  | Staggered alignment | #4 | 1.40 | 1.60 | 1.14 | 1.00 | 6.80 | 2412 | 609.83 |
| **WF-AJP 4L** | Direct alignment | #5 | 1.00 | 1.70 | 1.70 | 0.95 | 5.52 | 3665 | 494.11 |
|  | Staggered alignment | #6 | 0.70 | 1.16 | 1.66 | 0.95 | 3.80 | 4296 | 611.90 |

**Note:** ***NA*** values indicate that the in-plane electrical resistance exhibits extreme anisotropy, rendering the conductivity calculation invalid due to functional failure of the printed film. Each entry (#1 – #6) corresponds to one independently printed sample.


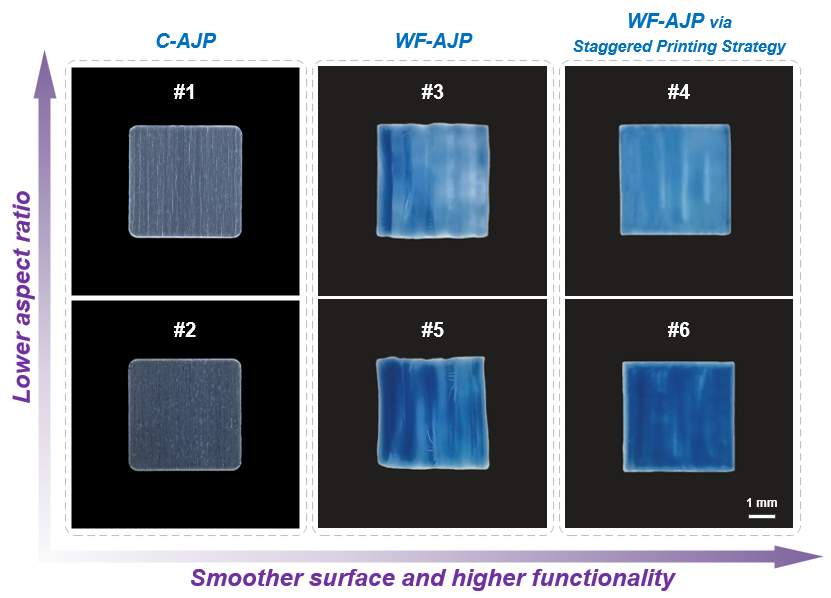


Figure S11. Comparison of film morphology across different printing strategies. Optical images of PEDOT:PSS films printed via C-AJP (left), WF-AJP (middle), and WF-AJP with a staggered printing strategy (right). The overall trend from left to right illustrates the evolution toward higher smoothness and functionality, while the vertical comparison reflects aspect ratio reduction.


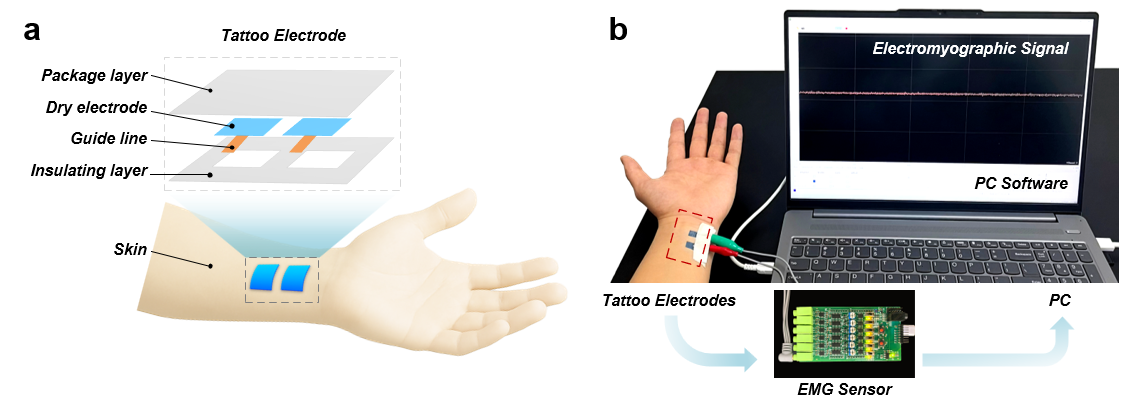


Figure S12. Integration and signal acquisition of printed tattoo electrodes for electrophysiological sensing. (a) Schematic illustration of the multilayer structure of the tattoo electrode, from top to bottom: the package layer provides environmental protection, the dry electrode enables electrical signal acquisition, the conductive guide line ensures signal transmission, and the insulating layer isolates the circuit from direct skin contact. The entire stack is laminated onto the skin, allowing for flexible and intimate epidermal integration. (b) Demonstration of electromyographic (EMG) signal acquisition using tattoo electrodes worn on the forearm. Signals are transmitted via an EMG sensor and displayed in real-time through PC software.


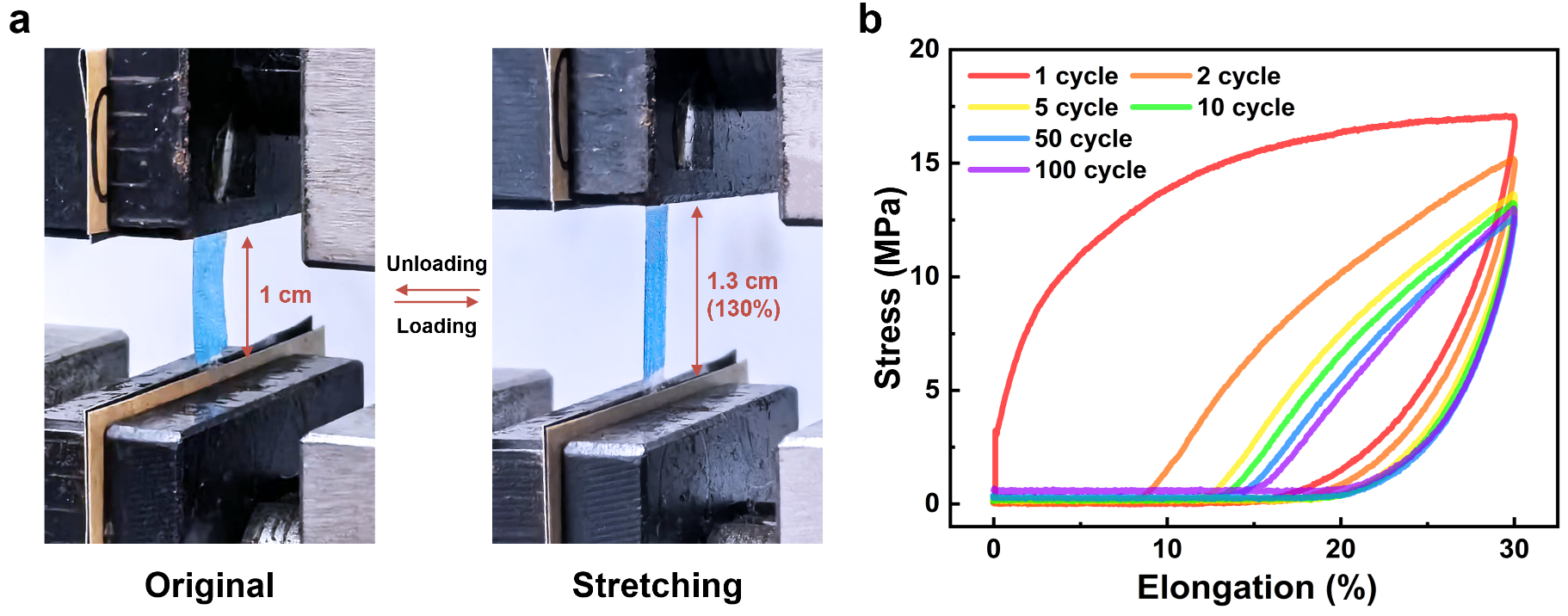


Figure S13. Cyclic mechanical durability of WF-AJP PEDOT:PSS tattoo electrodes. (a) Optical images of a representative sample (20 × 5 mm) under tensile loading, showing elongation from the original 1.0 cm to 1.3 cm (130%). (b) Stress-strain curves of the films under 1, 2, 5, 10, 50, and 100 cycles at 30% strain.

Supplementary Movie 1. Comparison of wide-flow and conventional aerosol jet printing processes.

It demonstrates the differences in aerosol jet morphology and deposition characteristics between WF-AJP and conventional AJP.

Supplementary Movie 2. Staggered printing strategy of wide-flow aerosol jet printing.

It illustrates the staggered deposition approach, in which the second printing layer is laterally offset from the first, effectively filling inter-track gaps and suppressing thickness nonuniformity caused by direct layer stacking.

Supplementary Movie 3. Wide-flow aerosol jet printed tattoo electrode functional presentation.

It shows the conformal adhesion, mechanical stability, and bioelectrical sensing capability of WF-AJP printed tattoo electrodes under dynamic skin deformation, demonstrating their potential for wearable and skin-integrated electronics.
